# Intelligent Recognition Using Ultralight Multifunctional Nano-Layered Carbon Aerogel Sensors with Human-Like Tactile Perception

**DOI:** 10.1007/s40820-023-01216-0

**Published:** 2023-11-09

**Authors:** Huiqi Zhao, Yizheng Zhang, Lei Han, Weiqi Qian, Jiabin Wang, Heting Wu, Jingchen Li, Yuan Dai, Zhengyou Zhang, Chris R. Bowen, Ya Yang

**Affiliations:** 1 CAS Center for Excellence in Nanoscience, Beijing Key Laboratory of Micro-Nano Energy and Sensor, Beijing Institute of Nanoenergy and Nanosystems, Chinese Academy of Sciences, Beijing, 101400 People’s Republic of China; 2 Tencent Robotics X, Shenzhen, 518054 People’s Republic of China; 3 School of Nanoscience and Technology, University of Chinese Academy of Sciences, Beijing, 100049 People’s Republic of China; 4 Department of Mechanical Engineering, University of Bath, Bath, BA2 7AK UK; 5 Center on Nanoenergy Research, School of Physical Science and Technology, Guangxi University, Nanning, 530004 People’s Republic of China

**Keywords:** Multifunctional sensor, Tactile perception, Multimodal machine learning algorithms, Universal tactile system, Intelligent object recognition

## Abstract

Humans can perceive our complex world through multi-sensory fusion. Under limited visual conditions, people can sense a variety of tactile signals to identify objects accurately and rapidly. However, replicating this unique capability in robots remains a significant challenge. Here, we present a new form of ultralight multifunctional tactile nano-layered carbon aerogel sensor that provides pressure, temperature, material recognition and 3D location capabilities, which is combined with multimodal supervised learning algorithms for object recognition. The sensor exhibits human-like pressure (0.04–100 kPa) and temperature (21.5–66.2 °C) detection, millisecond response times (11 ms), a pressure sensitivity of 92.22 kPa^−1^ and triboelectric durability of over 6000 cycles. The devised algorithm has universality and can accommodate a range of application scenarios. The tactile system can identify common foods in a kitchen scene with 94.63% accuracy and explore the topographic and geomorphic features of a Mars scene with 100% accuracy. This sensing approach empowers robots with versatile tactile perception to advance future society toward heightened sensing, recognition and intelligence.

**Supplementary Information:**

The online version contains supplementary material available at 10.1007/s40820-023-01216-0.

## Highlights

- The tactile performance of ultralight multifunctional sensors can reach the level of human tactile perception.
- An individual sensor can provide multiple tactile sensations: pressure, temperature, materials recognition, and 3D location. Therefore, it is no longer necessary to integrate multiple sensing modules with different functions, which greatly simplifies system complexity and reduces energy loss.
- The tactile system with multimodal learning algorithms has universality and can accommodate object recognition tasks in various application scenarios (e.g., Mars and Kitchen).

**Supplementary Information:**

The online version contains supplementary material available at 10.1007/s40820-023-01216-0.

## Graphical abstract

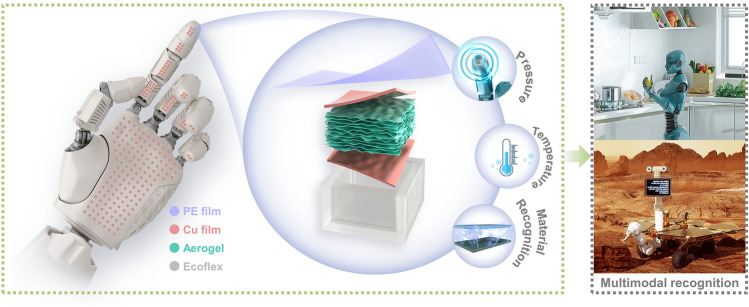

## Introduction

The human body is a masterpiece of nature's ingenuity, with a wealth of sensory organs to perceive our world [1–4]. A key factor to the advanced perception of a range of external stimuli is multisensory fusion, which allows humans to interact with the surrounding environment flexibly and identify objects precisely. Replicating this ability in robots to provide to multifunctional object recognition represents a significant challenge [5]. In general, robot vision technologies have made substantial progress in rapidly recognizing objects [2, 6–10], and some have even been commercialized. However, vision systems are often accompanied by complex and bulky equipment. In addition, in specific environments such as in the dark, in visual blind spots (corners, gaps, and obstructions) [7, 11]; or in extreme weather conditions such as in a haze, dust storms, fog, and heavy rain, the accuracy of the visual recognition function can decrease dramatically, or even fail completely. As a result, there is a critical need to develop portable and multifunctional tactile systems for robots that can compensate for the deficiencies of vision systems.

Multifunctional tactile systems have been used to assist robots in object recognition [12–16]. However, the use of conventional unimodal machine learning algorithms leads to limitations in recognition capability in complex scenes. Other researchers have developed haptic systems with multimodal algorithms, that combine complementary information, to perform recognition tasks in challenging environments [11, 17, 18]. While this approach can take full advantage of the multiple sensing modes of haptic systems and achieving more robust inference results, it often requires sophisticated integration of multiple sensing modules to attain multifunctional characteristics, leading to partitioned sensing and identification. Therefore, there is a need to integrate versatility within individual sensing units [19] to provide full spatial resolution for the robot. Table S1 provides a detailed comparison of the sensing performance, algorithm architecture, and object recognition capabilities of recently reported multifunctional tactile systems. From the material point of view, some researchers have prepared multifunctional sensor devices based on nanomaterials such as nanowires and MXene [20–23]. However, the multifunctional sensing capabilities of these sensors are hardly comparable to the perception of human skin. Thus, it is difficult to apply it to actual intelligent scenarios such as robotics and artificial intelligence. However, nano-layered carbon aerogel materials have great advantages in multifunctional sensing properties and smart applications. The nano-layered microstructure provides excellent mechanical, piezoresistive and electrical properties of the sensor. Thereby, the device can perceive pressure and temperature like human skin. In addition, aerogel is an extremely low-density material. Therefore, aerogel-based sensors have a very small weight, which is more conducive to flexible integration and wearability in actual applications. An ability to reduce the size and weight of individual sensors and constructing universal multimodal algorithms are also vital factors to establish a widely applicable multifunctional haptic recognition platform. More interestingly, the multifunctional haptic platform is universal and can be applied to different intelligent scenarios. In daily life, healthy, nutritious and tasty meals are vital for everyone, but cooking by yourself is very time and energy-consuming. Therefore, the development of a kitchen robot that can cook autonomously is a current research hotspot. Among them, independently distinguishing and selecting ingredients is the difficulty in developing a new generation of kitchen robots [24–26]. Therefore, the aid of precise tactile recognition capabilities will provide new ideas in the field of kitchen robots. Furthermore, during the exploration of Mars, there are urgent international challenges to explore the information on topography and landforms and to identify water sources [27–35]. Therefore, it is an innovative and compelling solution to endow the Mars rover with multifunctional tactile recognition capabilities.

**Fig. 1:**
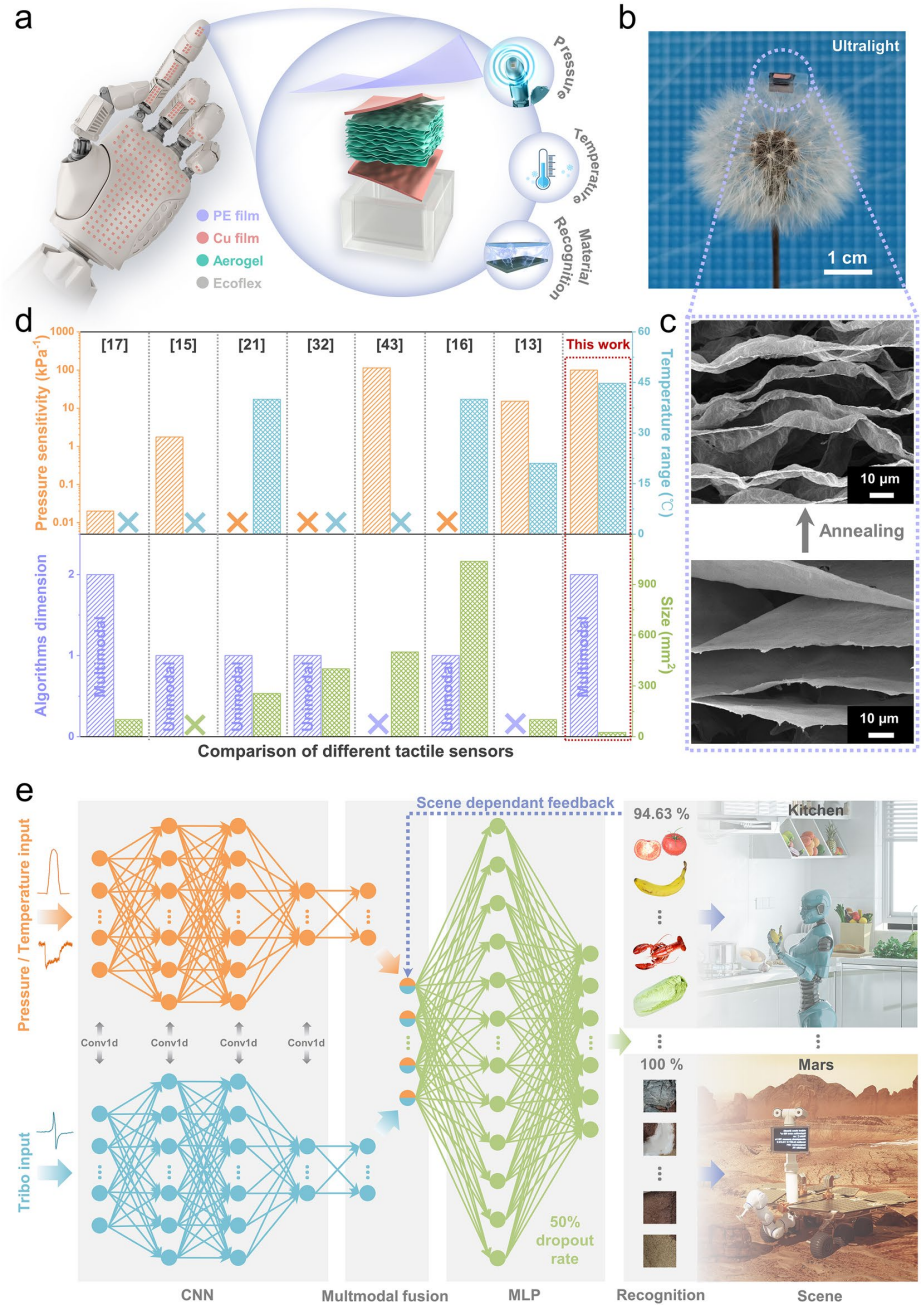
Concept and design of a multifunctional tactile system for intelligent identification. **a** A conceptual diagram of the MTAS sensing system integrated into a robot hand to provide robots with haptic perception capabilities. Right: hierarchical structure and multifunctional sensing features of MTAS. From top to bottom, the polyethylele (PE) electrification layer, the upper electrode, the rGO/CNCs nano-layered carbon aerogel, the lower electrode, and the Ecoflex elastic support layer. **b** Photograph of the ultralight MTAS floating on a dandelion. **c** Scanning electron microscopy (SEM) images of the nano-layered carbon aerogel with an ordered structure. **d** Comparison of the pressure sensitivity, temperature detection range, algorithm dimensions, and sensor size of the MTAS and previously reported state-of-the-art tactile sensors. **e** Multimodal supervised learning algorithm architecture and application scene of MTAS haptic system. The algorithm shows excellent robustness and universality, applying to kitchen, space applications, and more intelligent scenarios

In this article, we combine a multifunctional tactile nano-layered aerogel sensor (MTAS) and a multimodal deep learning algorithm to establish a universal haptic platform for robots (Fig. 1). At the front end of the system, a flexible MTAS is integrated into the surface of the robot to collect a variety of sensing signals (Fig. 1a). At the back end of the system, a multimodal deep learning algorithm is able to generalize the fusion of signals to recognize objects with minimal modification (Fig. 1e). Via this approach, robots can achieve human-like tactile perception to accurately identify objects in intelligent scenes without relying on the visual conditions.

## Experimental Section

### Materials

Graphene oxide (GO) powders, product number XF002-2, were purchased from Nanjing XFNANO Materials Tech Co., Ltd. Cellulose nanocrystals (CNCs, suspension, 8.0 wt%) were purchased from Guilin Qihong Technology Co., Ltd. The silicone elastomer (Ecoflex 0020), used with a 1:1 ratio of part A to part B, was purchased from Shanghai Smarttech Co., Ltd.

### Preparation of Nano-layered Carbon Aerogel

The nano-layered carbon aerogel was manufactured as follows: (i) Preparation of GO/CNCs suspension. Firstly, the GO suspension (1 mg mL^−1^) was prepared by dispersing the GO powder in distilled water, stirring thoroughly, and sonicating until the GO powder was well dispersed without precipitation. Then, the CNCs (8.0 wt%) were added to the GO suspension according to a 1:4 solid-content-ratio of GO to CNCs, and a homogeneously dispersed GO/CNCs suspension was obtained by thorough stirring and sonication. (ii) A pre-designed 3D-printed mold (Fig. S2b, individual mold size: 10 mm × 10 mm × 5 mm) was fixed to the side wall of a steel box (Fig. S2c and Note S1). Using a pipette gun, 0.5 mL of the GO/CNCs suspension was injected into the mold separately to avoid introducing air bubbles. (iii) Liquid nitrogen was quickly poured into a steel box and directed to freeze the GO/CNCs suspension for 30 min. The GO/CNCs ice crystals were formed when the GO/CNCs suspension in the mold was completely frozen. (iv) The GO/CNCs ice crystals were placed in a freeze dryer for 3–5 days to the obtain GO/CNCs aerogel (size: 10 mm × 10 mm × 5 mm). (v) The aerogel was annealed in a tube furnace under an N_2_ atmosphere. First, the temperature was increased from room temperature to 200 °C at a rate of 5 °C min^−1^ and maintained for 2 h; then, the temperature was increased from 200 to 700 °C at a rate of 3 °C min^−1^ and maintained for 2 h [36]; finally, a rGO/CNCs nano-layered carbon aerogel (size: 9 mm × 9 mm × 4 mm) was obtained after natural cooling.

### Fabrication of the Multifunctional Tactile Nano-layered Aerogel Sensors

First, the rGO/CNCs nano-layered carbon aerogel was cut into cubes with dimensions of 4 mm × 4 mm × 2 mm (Fig. S2e–g). Second, part A and part B of the Ecoflex elastomer were added to the beaker in a 1:1 ratio and mixed sufficiently. After draining any air bubbles, the uniformly mixed Ecoflex was poured into the mold. Then, the Ecoflex was cured in an oven at 80 °C for 2 h. After cooling down and demolding, the flexible supporting layer of the multifunctional sensor was produced (external size: 5 mm × 5 mm × 3 mm). Third, as the upper and lower electrodes of the multifunctional sensor, copper sheets were cut to 4 mm × 4 mm and connected to the wires with conductive silver paste. Fourthly, a polyethylene (PE) film was cut into 10 mm × 10 mm and used as a triboelectric layer for the multifunctional sensor. Fifth, one copper electrode was fixed to the Ecoflex supporting layer to act as the sensor’s lower electrode, and the other copper electrode was fixed to the center of the PE triboelectric layer to act as the sensor’s upper electrode. Sixth, the PE triboelectric layer with the upper electrode, nano-layered carbon aerogel, and Ecoflex support layer with the lower electrode was assembled, and the interface between the PE electrification layer and Ecoflex support layer was firmly bonded by acrylic glue. The sensor was then fully encapsulated to protect the nano-layered carbon aerogel from the external environment. Finally, a multifunctional nano-layered carbon aerogel tactile sensor with a core functional area of 5 mm × 5 mm × 3 mm was prepared.

### Characterization and Measurements

The morphology of GO/CNCs aerogels and rGO/CNCs carbon aerogels were characterized by field emission scanning electron microscopy (FEI, Nova NanoSEM 450) at a 5 kV acceleration voltage. Evaluation of the multifunctional sensing performance was achieved with a system consisting of a mechanical testing stage (IMADA, MX2-500N) and a force gauge (IMADA, ZTA-5N). An infrared thermographic camera (PI400, Optris) was used for temperature monitoring. The electrical signal of MTAS was measured with a digital source meter (Keithely, 2611B). The integration of the 7-axis robotic arm (SIASUN, SCR3) and the 6-axis force/torque transducer (ATI, 9105-TW-Nano43-R-5-EC8) provided a stable measurement environment for intelligent recognition systems in multiple scenarios.

### Multifunctional Sensing Signals Acquisition

Figure S3a shows the test circuit diagram, acquisition mode and output signals for multifunctional sensing (pressure, temperature, triboelectricity). Pressure: The pressure sensing performance test collected current signals at a bias voltage of 0.1 V through a dual-electrode mode. Temperature: The temperature sensing performance test collected current/voltage signals in self-powered mode via dual-electrode. Triboelectricity: The triboelectric sensing performance test collected voltage signals in self-powered mode through a single-electrode. The sensing characteristics were systematically assessed by loading a 1 GΩ resistor in the external circuit. We selected a fluorinated ethylene propylene (FEP) film as the test material for the fundamental triboelectric performance (PE was used as the triboelectric layer for MTAS). In summary, the collected multifunctional signals are input into a multimodal supervised learning algorithm for object classification and recognition.

### Multimodal Machine Learning Approach

Since our data was a time-variant signal, 1D convolution was used to extract feature. ReLU was used as the activation function. Max pooling layer was used to down sample the feature map. By stacking the above operations, the signal features were extracted. As shown in Fig. S4, the temperature/pressure signal and triboelectric signal were processed by the similar network with different neuron numbers per layer due to the different signal dimensions. During the fusion process, the extracted temperature/pressure feature map and the extracted triboelectric feature map were concatenated and fed into a fully connected neural network with three layers for classification. The dropout layer randomly sets input units to 0 with 0.5 rate at each step during training time, which helped to prevent overfitting. This enhanced the generalization ability of the neural network for scalable sensory data fusion, and considered the noise present in the signal.

During the training stage, we sampled 64 data as one batch. At each training step, we sampled a batch size of data to update the network parameters. An Adam algorithm was used as the optimizer to update the network parameters. The learning rate was set to 5 × 10^–4^ for the Adam optimizer and the weight decay was set to 1 × 10^–5^ to prevent overfitting; the hyperparameters could be slightly different in different scenarios. The cross entropy loss was used as the loss function and the Epoch number was set to 200, which means the learning algorithm will work through the entire training dataset 200 times. For each epoch, the network was evaluated with recognition accuracy on the test set. The network was well trained and convergent after 200 epochs and the recognition performance remained stable.

### Unimodal Machine Learning Approach

We implemented two unimodal learning approaches to classify objects. Initially, the sensor signal such as pressure/temperature and triboelectric voltage were processed through several convolutional layers to extract feature maps, with a polling layer to down sample the feature maps. Subsequently, the output of the CNN was connected to a fully-connected neural network with three layers, to classify the object’s type. For the temperature-only/pressure-only network, the triboelectric extraction CNN network was removed and only the temperature/pressure feature map was connected to the three fully-connected layers to classify objects. For the triboelectric-only network, the temperature/pressure extraction CNN network was removed, in a similar manner to the temperature-only/pressure-only network.

## Results and Discussion

As an important sensing element, the MTAS exhibits three remarkable advantages of, (i) sensing multifuncitonality, (ii) an ultra-lightweight nature and (iii) universality of application. Based on an ultralight reduced graphene oxide/cellulose nanocrystals (rGO/CNCs) carbon aerogel, the MTAS simultaneously provides both sensing multifunctionality and an ultralight nature, two generally mutually competitive features that are vital for sensing applications. In terms of multifunctionality, an individual MTAS is able to sense pressure, temperature and triboelectricity independently. As a result, it is no longer necessary to integrate multiple sensing modules on a robot surface, which greatly simplifies system complexity and reduces energy loss [4, 11, 12, 17, 37]. In terms of an ultra-lightweight mass, the MTAS can readily float on a dandelion (Fig. 1b) and its weighs only 64.5 mg (Fig. S2a). Based on the ultralight MTAS platform, the mass of a robot tactile system can be dramatically reduced, which is beneficial for large-scale integration. In terms of universality, MTAS tactile system has been applied to the home (kitchen) and space (Mars) scenes, demonstrating its prominent universality and application potential for a range of intelligent scenarios.

### Design of Multifunctional Tactile Nano-layered Aerogel Sensor Haptic System for Intelligent Identification

The right image of Fig. 1a shows the hierarchical structure of the MTAS, where detailed preparation details are described in “Methods”. The aim of the larger upper area of the polyethylene (PE) electrification layer is to provide superior material identification capability. The wavy layered rGO/CNCs carbon aerogel is a multifunctional sensing core. Figure 1c shows the microscopic morphology of the rGO/CNCs nano-layered aerogel with a wavy layered ordered structure because of the unique directional freezing and annealing technique (Note S1 and Fig. S2b–g). Because annealing leads to inhomogeneous stress distribution in the flakes, which induces wrinkling of relatively flat GO/CNCs flakes (the bottom of Fig. 1c) into rGO/CNCs flakes with a folded wavy morphology (the top of Fig. 1c). From a mechanical standpoint, the wavy lamellar structure after crumpling can provide a more stable elastic structure. Thanks to the stress can be well-dispersed out-plane, and the carbonized CNC gives stronger interconnections between the rGO layers, ensuring the stability of the in-plane structure [36]. Subsequently, the microstructure of nano-layered aerogel in different orientations and excellent mechanical properties are analyzed in depth, see Note S1 and Figs. S1–S2. Copper films are used to provide electrical connections. The lower Ecoflex elastomer support layer is molded into a hollow box shape, which together with the PE layer, completely encapsulates the sensing core, providing all-around protection and support for the nano-layered carbon aerogel from the external environment interferences. In addition, the MTAS acquires multifunctional signals in different acquisition modes; see Method and Fig. S3a. The signals can be easily decoupled and the logic of decoupling multifunctional signals is shown in the flow chart (Fig. S3b).

Figure 1d demonstrates the significant advantages of the MTAS in all aspects of multifunctional sensing performance, algorithm design, and device size [13, 15–17, 37–39]. Overall, MTAS has the best multifunctional sensing performance, demonstrating high sensing capability in both pressure and temperature. Multimodal machine learning algorithms can combine multiple dimensions of input data features, dramatically improving recognition accuracy and demonstrating outstanding universality in multiple recognition tasks. The small size of the individual sensing units provides high resolution for multifunctional tactile sensing.

The framework of multimodal machine learning algorithm (Figs. 1e and S4) includes two Convolutional Neural Networks (CNN) for extracting tactile information separately from time-variant tactile signals, and a three-layer fully connected neural network (0.5 dropout rate) which concatenates the extracted information for ultimate categorization. Compared to Very Deep Convolutional Networks, that assist in processing tactile information [11], our 1D Convolutional Network is much smaller in size and can be readily applied to devices with limited computational resources. Moreover, scene-related feedback is added to the learning framework to explicitly adjust the weight ratio of multifunctional signals (Note S2), ensuring a high recognition rate in challenging scenes. The multimodal learning framework was successfully applied to a kitchen home scene and Mars planet scene with 94.6 and 100% accuracy. This indicates that the robust architecture provides MTAS tactile system with excellent universality to handle wider range of object recognition tasks. Furthermore, on comparing our approach with unimodal learning methods (see Methods), the multimodal approach obtained more accurate recognition results, verifying the importance of multimodal fusion.

### Pressure Sensing Property and Mechanism of MTAS

**Fig. 2:**
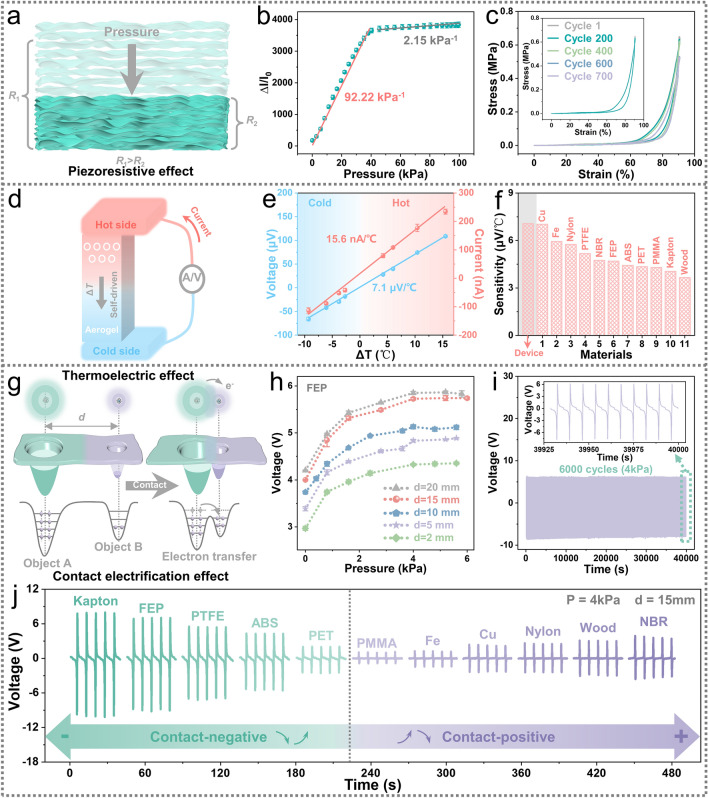
Multifunctional sensing performance and mechanism characterization of MTAS. **a** Schematic of the piezoresistive effect. **b** Sensitivity of pressure sensing and normalized current response (relative current, ΔI/I_0_) at different pressures. Error bars represent the standard deviations based on three tests. **c** Stress–strain curves of MTAS at 90% compressive strain for 700 cycles. Inset: stress–strain curves for the first and 200^th^ cycles. **d** Schematic of the thermoelectric effect. The thermoelectric effect can convert a ΔT into an electrical signal, implementing self-driven temperature sensing. **e** Voltage and current response of temperature sensing at different ΔT (the corresponding T_c_ is the range of 21.5–66.2 °C). The temperature sensitivity was calculated by a linear fit with a voltage sensitivity of 7.1 μV/°C and a current sensitivity of 15.6 nA/°C. Error bars are s.d. from three tests. Real-time temperature is monitored by an infrared camera throughout. **f** The histogram of the heat transfer properties of 11 materials (Cu, Fe, Nylon, PTFE, NBR, FEP, ABS, PET, PMMA, Kapton, and Wood). Size: 10 mm × 10 mm × 1 mm. **g** electron-cloud-potential-well model based on the contact electrification effect. **h** Influence of contact pressure and maximum separation distance (d) on the triboelectric performance. Error bars are s.d. from five samples. **i** Robustness testing of MTAS triboelectric sensing performance. **j** Triboelectric signals characteristic of 11 materials. Strictly controlled experimental variables (contact force 0.1 N, pressure 4 kPa, contact separation speed 300 mm min.^−1^, d = 15 mm, room temperature ~ 22 °C, humidity ~ 33%) and used anhydrous ethanol to remove the effect of the initial surface charge before testing. Inset: Electronegativity ranking of the 11 tested materials (the same regularity was given three times on different days)

Figure 2a illustrates the pressure sensing mechanism that is based on the piezoresistive effect. The conductive wavy multilayer skeleton provides the rGO/CNCs nano-layered carbon aerogel with a high degree of spatial compressibility. On applying pressure to the system, in Fig. S5a, the conductive skeleton is compressed and becomes more compact, thereby producing additional conductive pathways in the MTAS (Fig. 2a). As a result, the MTAS resistance decreases with an applied compressive load, and the detection current increases. The current–voltage (*I–V*) curves exhibit a typical linear correlation (Fig. S5b), indicating that the MTAS forms a favorable ohmic contact. With increasing pressure, the slope of the *I–V* curve gradually increases, which implies a decrease in resistance and echoes the mechanism outlined in Fig. 2a.

From Fig. 2b, the response of the MTAS is in the pressure range of 0.04–100 kPa is superior to the human tactile perception [17, 24]. This means that the MTAS can operate at pressures over 3873 times its own weight, thereby exhibiting a stress resistance comparable to that of an ant colony [25]. In addition, the curve is divided into two stages, a first stage (below 40 kPa), where the normalized current increases dramatically, and an excellent pressure sensitivity of 92.22 kPa^−1^ is achieved (Note S3). In the second stage (above 40 kPa), the relative current is gradually saturated as the microstructure is fully compacted. During this high-pressure phase, the Ecoflex elastomer support layer shares most of the pressure for aerogels, preventing the collapse of the nano-layered aerogel microstructure and any other damage. Moreover, owing to the resolution limitation of the force gauge, the minimum detectable force of MTAS is 0.04 kPa (Fig. 2b). Thus, in Fig. S5d and Note S4, four actual objects were selected to investigate the minimum pressure that MTAS can detect in daily life, a 117 mg (~ 30 Pa) Vitamin C pill. The response time and recovery time of the MTAS are 11 and 14 ms (Fig. S5c), indicating that the MTAS can respond to pressure at the millisecond level, much like human haptics [17, 19].

The stress–strain curve of the MTAS at a high strain of 90% for 700 compression cycles, exhibits a typical crescent shape; see Fig. 2c. In addition, the narrower hysteresis return line indicates that the MTAS has a small energy dissipation during a compression cycle. In particular, the stress–strain curve after 200 cycles (Fig. 2c inset) is almost indistinguishable from the initial curve, demonstrating the excellent fatigue resistance of the MTAS. Finally, the cyclic stability of the MTAS is demonstrated in Note S5.

### Performance and Mechanism of MTAS for Self-powered Temperature Sensing

The temperature sensing mechanism of MTAS is based on the thermoelectric effect (Fig. 2d), since a homogeneously dispersed electrically conductivity rGO component in a nano-layered carbon aerogel provides excellent thermal and electrical properties [26, 40]. The temperature difference between the upper and lower surfaces of the MTAS drives inner carriers to move from the hot side to the cold side, and the temperature difference is converted into an electrical signal. Therefore, the MTAS can sense temperature in a self-powered way, since no external electrical energy source is necessary. We constructed a system to evaluate the temperature sensing performance of the MTAS (see Fig. S6a and Note S6). The MTAS is able to sense temperature sensitively in both voltage and current modes (Fig. 2e) and the corresponding temperature profiles and their real-time infrared images (Fig. S6c) indicate that the MTAS can detect temperatures in the range of 21.5–66.2 °C. Theoretically, a wider temperature detection range is possible (Note S7). The safe range tolerated by human hand is approximately 20–50 °C [41, 42], indicating that the MTAS has a temperature sensory range comparable to human touch. Note S7 provides additional details on temperature sensing capabilities.

Furthermore, the temperature sensing function can distinguish materials with different heat transfer characteristics (Fig. 2f). According to the evaluation system (Note S8 and Fig. S7a), we tested the heat transfer properties of 11 lamellar materials (Figs. S7–S12). We calculated the heat transfer sensitivity of materials and compared it with an individual MTAS device. A single device displayed the highest sensitivity since direct contact reduces heat loss. Metals such as Cu and Fe have the highest sensitivity among the 11 materials tested, which corresponds to their superior thermal conductivity. Polymeric materials are located in the middle range of sensitivity. Due to the presence of a large number of hollow structures, wood has the lowest temperature sensitivity, due to the poor heat transfer performance and makes it an ideal material for thermal insulation [43]. Therefore, a MTAS can identify material types by judging the heat transfer properties of the material itself.

### Systematic Evaluation of the Material Identification Characteristics of MTAS

Based on the contact electrification effect (Fig. 2g and Note S9) which is prevalent between various interfaces in nature [44, 45], the MTAS is able to collect triboelectric signals from various free-moving objects by utilizing a flexible material identification function. The triboelectric mechanism of operation of a MTAS during a contact-separation cycle is shown in Fig. S13a and Note S10. The triboelectric signals gradually improve as the contact pressure increases until it reaches saturation at 4 kPa (Figs. 2h and S14), with the same overall response for all maximum separation distances (d). Consequently, a contact pressure of 4 kPa (0.1 N) was implemented for all subsequent triboelectric tests, both for safety reasons and for achieving relatively high output signals. Notably, due to the fully encapsulated design, the MTAS has excellent triboelectric stability, with no performance loss in 6000 cycles tested at 4 kPa pressure (Fig. 2i), thereby demonstrating the outstanding robustness of the MTAS. Further triboelectric performances are characterized in Fig. S13b–d and Note S11.

Examination of the triboelectric function in more detail, the MTAS can gather the specific triboelectric signals generated by objects based on their ability to gain or lose electrons, including characteristic information such as signal direction, amplitude and waveform [13, 38, 46]. We selected 11 materials (Fig. S13e) for triboelectric testing. The characteristic waveforms (Fig. 2j) of five materials are from negative to positive, while other six materials are from positive to negative. The amplitudes of triboelectric signals generated by the 11 materials are also different. This is attributed to the difference in electronegativity between the polyethylene (PE) triboelectric layer and the materials under test. The greater the difference in electronegativity, the greater amplitude of output signals [44, 45]. Accordingly, the electronegativity of the 11 materials is ranked in Fig. 2j. The material closer to the green (left) side is more electronegative relative to PE, while the material closer to the purple (right) end is more electropositive relative to PE, which is basically consistent with the triboelectric series [44, 45]. It is worth considering that the contact-based sensing method, and the specific triboelectric signals of objects, allow the MTAS to be utilized as a tactile recognition tool. Combining computer technologies, such as machine learning, can allow the multifunctional sensible capability to be applied to more intelligent scenarios.

### Multimodal Object Recognition System in Kitchen Scene

**Fig. 3:**
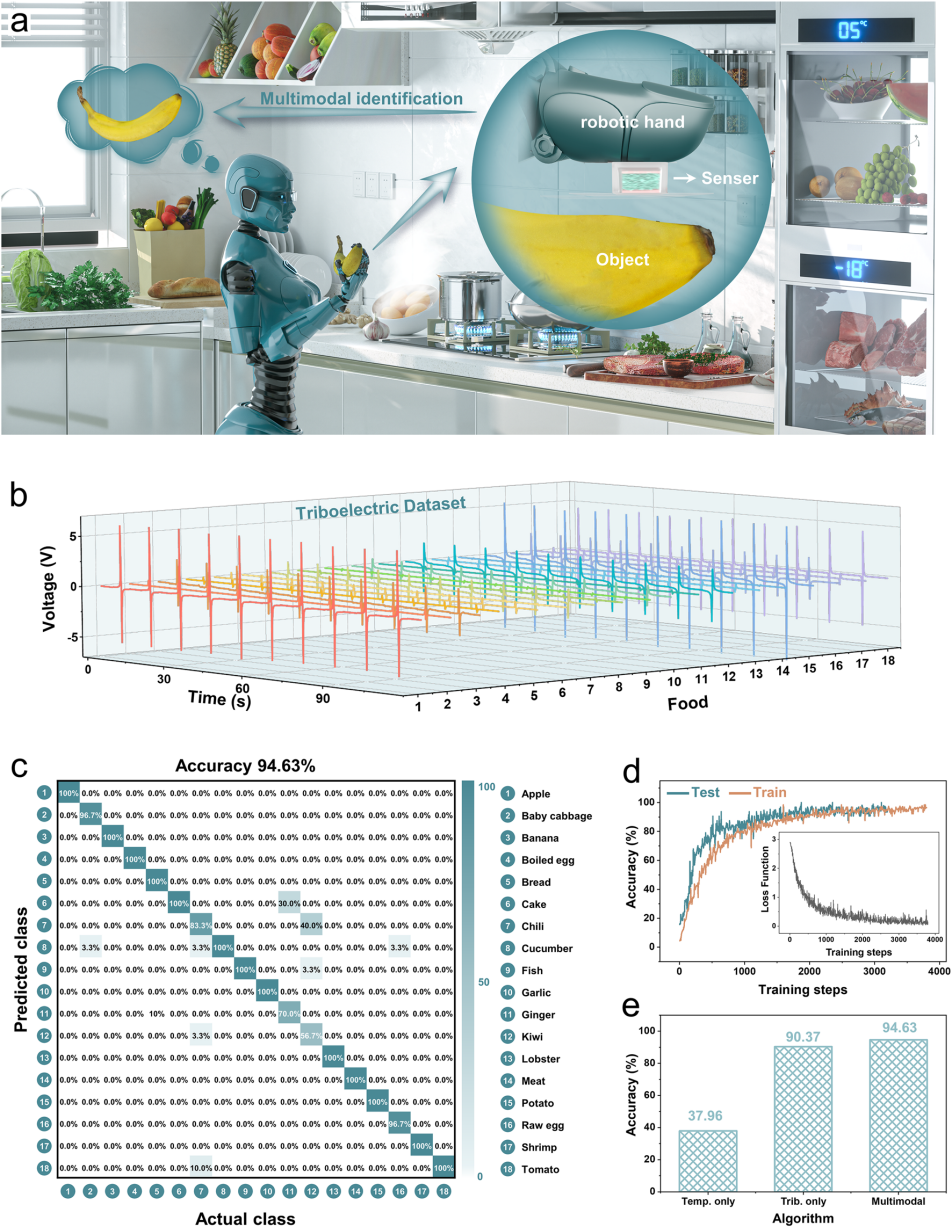
MTAS-based multimodal object identification system in kitchen (home) application. **a** Concept diagram of a kitchen robot integrated with MTAS intelligence system to identify food. **b** Triboelectric recognition dataset for 18 food (10 cycles). **c** Confusion matrix for multimodal identification of 18 foods in kitchen scenarios. **d** Variation curves of training and testing accuracy with training steps. Inset: Variation curves of loss with training steps. **e** Histogram of accuracy comparison between unimodal approach and multimodal approach

The MTAS can be applied to a kitchen scene in a home by combining it with a multimodal learning algorithm (Fig. 3a). This vividly demonstrates the logical concept of a kitchen robot that integrated with MTAS to recognize food by touch, which provides a viable approach for developing kitchen robots without human assistance (Note S12). The MTAS kitchen robot hand can independently distinguish and select ingredients based on its tactile recognition capabilities. This can reduce the workload in the kitchens and provide more customized services for humans. In particular, in kitchens with smoke and steam, the tactile recognition function of MTAS can circumvent the failure of traditional visual recognition system.

In order to maintain food freshness and prolong storage time, food is stored in different areas according to its appropriate storage temperature (Note S13). Thus, the robot can collect temperature information regarding a food surface when it grasps foods in the kitchen. Based on the above traits, we used the temperature sensing and material identification of the MTAS to achieve multimodal fusion recognition of food. We constructed a programmable test system for automating data acquisition, which can exclude artificially signal interference (Note S14), including the MTAS, robot arms and measured food (various types of representative foods in the kitchen, Fig. S15a and Note S14).

The multimodal learning dataset of 18 foods in a kitchen scene were collected by multipoint sampling (Note S15). This strategy captures more complete information regarding the food surface and provides the robot with more flexible food recognition capabilities. Figures 3b and S15b plot the triboelectric and temperature recognition signals for 10 cycles of 18 foods, respectively. The collected learning data is pre-processed (Note S16) and input into the neural network. Since the dataset contains data from different positions of food, we randomly select 70% positions as training data and 30% positions as testing data. In the training stage, we sample 64 data samples from dataset as a single batch, and update the network at each training step. The training and testing accuracy rate continually increases and the loss continues to decrease throughout 3000 steps and remains stable for a higher number of training steps (Fig. 3d). The confusion matrix indicates the final accuracy of 94.63% for multimodal recognition of 18 foods (Fig. 3c), and most food can be recognized with 100% accuracy. In addition, two unimodal learning approaches were implemented to classify 18 foods (Fig. S25 and Note S17), and both approaches have lower accuracy than our multimodal supervised learning approach. Figure 3e demonstrates the comparison of recognition results between the multimodal algorithm and two unimodal algorithms, which demonstrates that multimodal sensing and fusion can significantly improve recognition accuracy.

### Multimodal Object Recognition System in Mars Scene

**Fig. 4:**
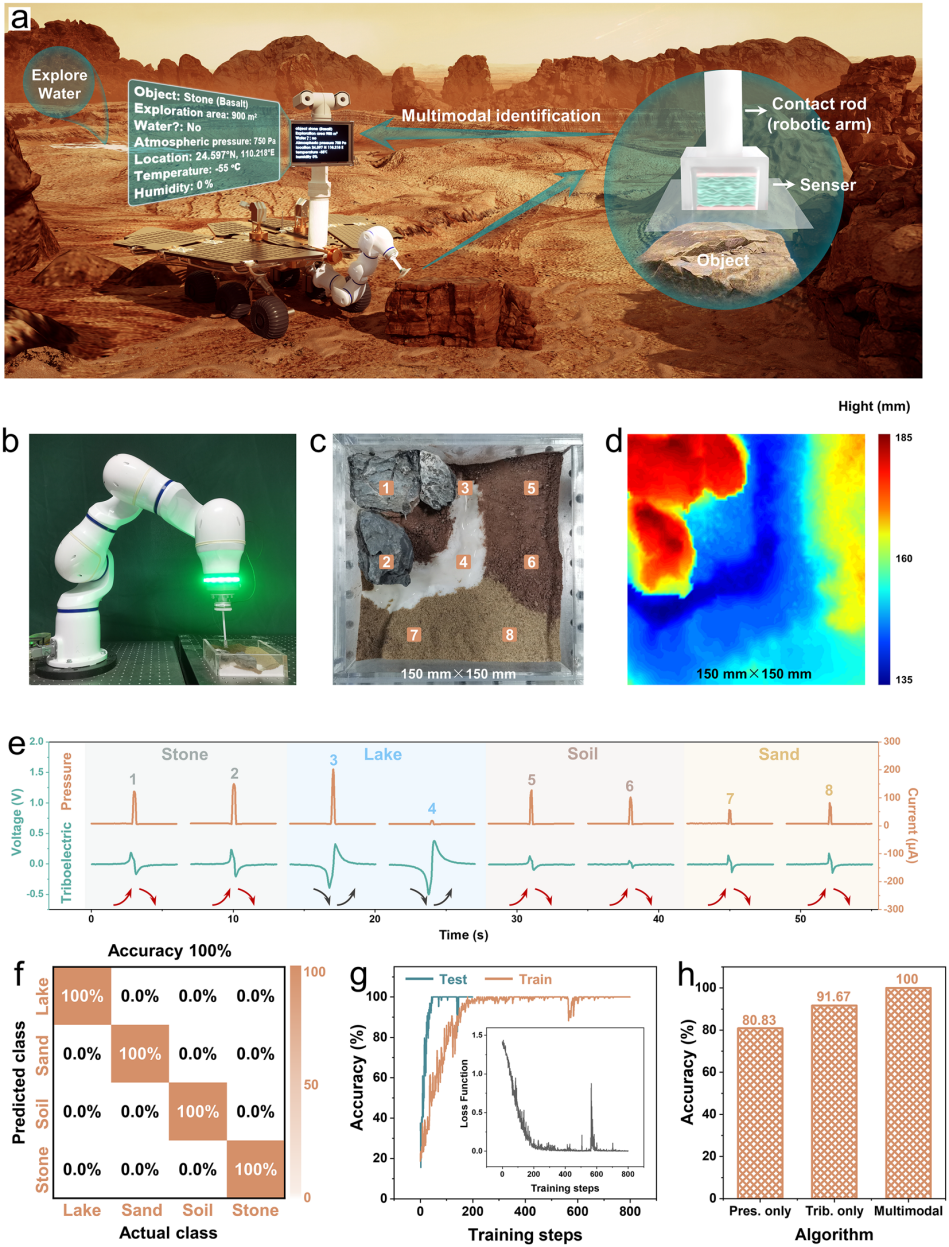
MTAS-based multimodal object identification system in Mars space application. **a** Conceptual diagram of a Mars rover integrated with MTAS intelligence system to identify Martian topography and landforms. **b** Photographs of an automated intelligent system for collecting machine learning data from a Mars micromodel. It consists of a seven-axis robotic arm, a six-axis force control device, a touch rod, MTAS, and a Mars micromodel. **c** A micromodel simulating the topographic and geomorphic features of Mars. The number markers represent the distribution of 8 feature points for micromodel landforms identification. The 8 feature points are evenly distributed. Feature points 1 and 2 correspond to rocks, feature points 3 and 4 correspond to rivers, feature points 5 and 6 correspond to soils, and feature points 7 and 8 correspond to sands. **d** Topographic reconstruction simulation map (top view, pixel points: 5184) of the Mars micromodel with Matlab curve fitting. **e** Characteristic signals for pressure identification and triboelectric identification of different landforms. **f** Confusion matrix for four types of landforms multimodal recognition in the Mars scene. **g** Variation curves of training and testing accuracy with training steps. Inset: Variation curves of loss with training steps. **h** Histogram of accuracy comparison between unimodal approach and multimodal approach

More fascinatingly, the MTAS haptic recognition platform can be integrated into a Mars rover to explore the topographic and geomorphic features of Mars by remote control (Fig. 4a). This provides an inspiring blueprint for exploration of a planet suitable for human settlement. Most areas of Mars are shrouded in suspended dust with low visibility since large-scale sandstorms, surface winds, fog and other extreme weather are also suddenly encountered [27, 28], which can lead to failures of visual identification systems. Hence, it is crucial to develop Mars rovers with tactile recognition, which are not affected by the extreme climate.

Based on the above idea, we built a test platform to simulate the exploration of Mars (Fig. 4b), and the MTAS was integrated into the contact rod at the front of a robot arm. We designed a Mars micromodel (Fig. 4c) to simulate the topography and landforms of Mars (mountains, rocks, land, deserts, and dry rivers), which refer to the real Mars environment (Note S18) [29, 30]. When exploring the topographic and geomorphic features, the topography of the micromodel is first reconstructed (Figs. 4d and S26a), then the sampling points (geomorphic feature points) are identified based on the 3D simulated map, thus identifying the landforms of the feature points by the multimodal learning algorithms (detailed process in Note S19). The multimodal learning dataset of four landforms of the micromodel was collected by multipoint sampling (Note S20). Figure 4e shows the signal waveforms of eight feature points for landform identification (Note S21), indicating that pressure and triboelectric signals of different landforms are distinguishable.

The multimodal recognition framework in Mars scene is analogous to that in the kitchen scene (Fig. S29), indicating that our algorithm can be readily generalized to different cases by fine-tuning the network and training (Note S22 and Table S2). Pre-processed learning data (Note S16) is input to the multimodal neural network and the dataset for the micromodel is similarly divided between training and test data in a 7:3 ratio. The learning process reaches convergence and remains stable after approximately 300 steps for the training dataset and 100 steps for the testing dataset (Fig. 4g). The confusion matrix for multimodal recognition of micromodel indicates a final accuracy of 100% (Fig. 4f), indicating that all landforms can be recognized correctly. In addition, unimodal learning approaches are also implemented to classification. The pressure-only network performed better when classifying stone, while the triboelectric-only network achieved higher accuracy when classifying lake, sand and soil (Fig. S26b–e and Note S23). The multimodal network fuses both information and obtains the highest accuracy for all objects (Fig. 4h), thus verifying the importance of multimodal sensing and fusion.

Notably, the MTAS demonstrates outstanding robustness and waterproofing (Note S24) as a result of the fully encapsulated structure. Moreover, the MTAS has a unique signal waveform with respect to contact with water (Fig. S32), which is completely different from the signals of other objects (Note S25). Therefore, the MTAS has the potential to explore water resources on Mars, which is an essential basis for humans to judge whether Mars is habitable. Although stable liquid water has yet to be found, there is much evidence for the presence of hydrous minerals, polar ice caps, and water activity [31–35]. Consequently, continuing the search for liquid water resources is a significant and compelling task for Mars exploration.

## Conclusions

This work can inspire further research directions that can focus on two potential trends. Firstly, the MTAS can be integrated into intelligent identification systems with a higher distribution density. This will contribute to a more complete tactile mapping for more accurate recognition. Secondly, Mars frequently has rampant sandstorms and sand, dust, and dirt often cover the solar panels or camera lenses of space vehicles, preventing them from powering themselves and transmitting data and images back to Earth. As dust particles are electrostatically charged, the MTAS and other triboelectric devices can utilize its triboelectric properties to clean surfaces, including solar panels and lenses.

In conclusion, we have designed an ultralight and multifunctional tactile nano-layered aerogel sensor (MTAS) based on a wavy-layered carbon aerogel, which is combined with a multimodal supervised learning algorithms to provide multiple tactile sensations to robots. The MTAS combines three remarkable merits of sensing multifuncitonality, an ultralight nature and universality of operation. The multifunctionality of pressure, temperature, and triboelectric sensing and ultra-lightweight nature (65 mg) are both important features for sensors. The MTAS has a human-like [17, 19, 24] pressure detection range (0.04–100 kPa) and response time (11 ms), and a pressure sensitivity of 92.22 kPa^−1^, with excellent triboelectric durability for 6000 cycles. The temperature sensing range (21.5–66.2 °C) is comparable to human hands [41, 42]. The MTAS is able identify the inherent properties of material, namely heat transfer characteristics and electron gain/loss capability. In addition, each MTAS can act as an independent sensing element to provide multifunctional sensing abilities, without the need to integrate different sensing modules.

We combined the MTAS with a task-independent universal training framework to provide robust object recognition results in both home (kitchen) and space (Mars) scenarios. The MTAS tactile system was able to identify 18 common foods with 94.63% accuracy and explored topographic and geomorphic features with 100% accuracy, with the ability to explore the resources of Mars. Our new approach reduces the complexity of traditional robotic haptic recognition systems, and empowers robots with versatile tactile perception and object recognition in low visibility conditions, thereby opening up extensive opportunities to develop future society toward heightened sensing, recognition and intelligence.

## Supplementary Information

Below is the link to the electronic supplementary material.

Supplementary file1 (PDF 8939 KB)
