# Supplementary material for: Intelligent Recognition Using Ultralight Multifunctional Nano-Layered Carbon Aerogel Sensors with Human-Like Tactile Perception
Source: Nanomicro Lett. 2023 Nov 9;16:11. doi: 10.1007/s40820-023-01216-0 (PMC10635924; doi:10.1007/s40820-023-01216-0)
Supplement: Supplementary file 1 — Supplementary file1 (PDF 8939 KB) [file 40820_2023_1216_MOESM1_ESM.pdf]

Supporting Information for

Intelligent Recognition Using Ultralight Multifunctional Nano-Layered Carbon Aerogel Sensors with Human-Like Tactile Perception

Huiqi Zhao[1, 3], Yizheng Zhang[2], Lei Han[2], Weiqi Qian[1, 3], Jiabin Wang[1, 5], Heting Wu[1], Jingchen Li[2], Yuan Dai[2, *], Zhengyou Zhang[2], Chris R. Bowen[4], Ya Yang[1, 3, 5, *]

[1] CAS Center for Excellence in Nanoscience, Beijing Key Laboratory of Micro-Nano Energy and Sensor, Beijing Institute of Nanoenergy and Nanosystems, Chinese Academy of Sciences, Beijing 101400, P. R. China

[2] Tencent Robotics X, Shenzhen 518054, P. R. China

[3] School of Nanoscience and Technology, University of Chinese Academy of Sciences, Beijing 100049, P. R. China

[4] Department of Mechanical Engineering, University of Bath, BA2 7AK, UK

[5] Center on Nanoenergy Research, School of Physical Science and Technology, Guangxi University, Nanning 530004, P. R. China

[*]Corresponding authors. E-mail: yuan.dai24@hotmail.com (Dai, Y.), yayang@binn.cas.cn (Yang, Y.)

Note S1 Unique Directional Freezing/Annealing Technique and the Mechanical Characteristics of MTAS

In the directional freezing technique, we first created spaced freezing molds (Fig. S2b), eliminating the mutual influence of adjacent ice crystal temperatures, which offers a more perfect microstructure for aerogels. In addition, the freezing mold is fixed to the side wall of a steel box with good thermal conductivity (Fig. S2c). The steel box is filled with liquid nitrogen to provide a single directional temperature gradient for the GO/CNCs suspension in the mold. It contributes to the directional freezing process to obtain ordered growth of layered GO/CNCs ice crystals. Then, freeze-drying, ice crystals are sublimated to produce ultra-lightweight GO/CNC aerogels. Subsequently, by annealing under N_2 atmosphere, GO is reduced to rGO, and CNCs are carbonized to amorphous carbon. During annealing carbonization, the local volume reduction of CNCs is much more pronounced than that of GOs, because CNCs lose more weight than GO [S20, S46].

Figure S1a, b show 3D schematic of the GO/CNCs aerogel and the SEM images taken in different orientations, demonstrating that GO/CNCs aerogels have a relatively flat microscopic multilayered ordered structure arranged in parallel along the vertical direction. By annealing, the aerogel shrinks from 10mm×10mm×5mm to 9mm×9mm×4mm (Fig. S2d, e). Figure S1c, d show the 3D schematic of the carbonized rGO/CNCs aerogel after crumpling and the SEM images taken in different orientations. The wave-shaped microscopic lamellae can be observed in the vertical lamellar direction as well as along the lamellar direction.

The rGO/CNCs aerogel has exceptional mechanical properties such as compressibility, elasticity and fatigue resistance [S20, S46]. Figure S2e-g depict the remarkable shear resistance of the aerogel, which can be easily cut to the desired size without structural collapse or functional breakdown. Additionally, we keep the size of MTAS (5mm×5mm×3mm) as small

as possible while ensuring multifunctional sensing functions, which is the smallest size of non-polymer modified aerogel sensors ever reported in the literature [S24, S46-S50]. This assists in improving the resolution of haptic perception and offers the possibility of large-area integration on the surface of robotic hands. Furthermore, the aerogel exhibits anisotropic mechanical properties because of its directionally grown and layered ordered structure. In contrast to the external force along the sheet direction, the force applied in the vertical sheet direction does not easily fragment the wavy interlayer structure, resulting in higher mechanical strength and more robust of aerogel. Consequently, all subsequent sensing performance tests were performed with the pressure operation in the vertical sheet layer direction.

Note S2 Scene Feedback Mechanism for Multimodal Algorithms

According to the practical application, when one input perception is severely disturbed or destroyed, the network can be adjusted to rely on the other perception signals by increasing their relative weights, guaranteeing a high recognition rate of objects in challenging scenes.

Note S3 Pressure Sensitivity of MTAS

Here, the pressure sensitivity is defined as $S = \delta\left(\Delta I / I_0\right) / \delta P$, where S is the pressure sensitivity， ΔI is the change of current, I_0 is the initial current of MTAS, P is the applied pressure. According to Fig. 2c, the slope of the curve indicates the pressure sensitivity of MTAS.

Note S4 Minimum Detection Pressure of MTAS

MTAS can effortlessly respond to pressure signals from ultralight objects such as, a 117 mg (~30 Pa) VC pill, a 178 mg red button, a 594 mg metal button, and an 823 mg miniature toy (Fig. S5d), demonstrating the ability of MTAS to detect smaller pressure units.

Note S5 Cyclic Stability of MTAS

MTAS was tested over 5,00 cycles under a maximum force of 100 kPa without performance degradation (Fig. S5e). In the event that the device is operated beyond this 100 kPa safety pressure, the lifetime of the equipment will be significantly shortened. MTAS can also work for more than 2000 cycles at a low pressure of 2 kPa (Fig. S5f). The current signal of MTAS at different pressures is displayed in Fig. S5g. The inset shows more clearly the pressure sensing signal for a single pressure cycle (compression-release).

Note S6 Temperature Sensing Evaluation System

Peltier temperature module applies the temperature change to the upper surface of MTAS (contact temperature, T_c), whereas the lower surface remains at device temperature (T_d). A temperature difference (ΔT) is generated between the upper and lower surfaces of MTAS, which drives the external circuit to output a current/voltage signal.

Note S7 Theoretical Temperature Detection Range and Other Temperature Characteristics

Despite the theoretical possibility of a wider temperature sensing range, the Peltier variable temperature module is limited by its inability to withstand higher voltages, resulting in the failure to provide more extreme surface temperatures for MTAS.

The temperature difference ΔT, voltage, and current curves at the corresponding temperature are also recorded in Fig. S6c. The temperature response time of MTAS is shown in Fig. S6b, with response time and recovery time of 378 ms and 5.3 s, respectively. Because of our requirement for high resolution, the size of MTAS is designed to be as small as possible, which means a relatively small addition of the thermoelectric material rGO to each device, thus affecting the response time. In conclusion, after considering the effects of resolution, sensitivity, response time, and size (area and thickness), we chose the device size of 5mm*5mm*3mm for the in-depth study.

Note S8 Heat Transfer Performance Evaluation System

We selected 11 lamellar materials (same size) for evaluation. Materials were placed between the Peltier temperature module and the inverted MTAS (Fig. S7a). Peltier module applies the temperature change to the surface of 11 materials (T_m), whereas the lower surface of MTAS remains at device temperature (T_d). ΔT_2 is generated between Tm and Td. Due to the different thermal conductivity of materials, the temperature and heat transfer rate delivered to MTAS surface are different. Therefore, the variation of electrical signals with ΔT_2 (sensitivity) can reflect the inherent difference in thermal conductivity of materials. Figures S7-S12 show the temperature sensing signals for different materials.

The heat transfer sensitivity of 11 materials was obtained by the slope of a linear fit (Figs. S7c and S8-S12b, e) as a parameter to judge the heat transfer properties of the materials.

Note S9 Contact Electrification Effect

Figure 2g plots a schematic of the electron-cloud-potential-well model based on the contact electrification effect. Before contact, different substances have independent electron clouds and single-well potential. Physical contact causes the electron clouds to overlap. The energy barriers are reduced, asymmetric double-well potential is formed, and electron transfer occurs [S36, S50, S51].

Note S10 Operation Mechanism of Triboelectric Sensing

The initial state i, FEP and MTAS are relatively stationary and at the maximum separation distance (d). Since the FEP is far away from MTAS, negative charges are induced on the Cu electrode to keep the system in electrostatic equilibrium and no charge transfer occurs in the circuit. ii: When the FEP approaches MTAS process, the electrode induces negative charges that gradually decrease, and electrons flow through the load to the ground. Until state iii, MTAS and FEP are completely contacted and the electrostatic equilibrium state is achieved again. iv: As the FEP continues to separate from MTAS, negative charges are gradually induced on the electrode and electrons flow from the ground to the Cu electrode via the load, accompanied by a reverse output signal. Until the maximum separation distance, a contact-separation cycle is completed.

Note S11 Detailed Performance of Triboelectric Sensing

The output signal gradually increases as d increases until it saturates at d of 15 mm. When d is 20 mm, the measured output signal increases very weakly relative to 15 mm and is negligible. Therefore, the d for all subsequent triboelectric tests was chosen to be 15 mm (Fig. 2h).

We explored the effect of contact separation frequency on the output voltage signal (Fig. S13b). As the frequency increases, the output signal increases linearly. MTAS triboelectric sensing has a response time of 33 ms and a recovery time of 162 ms (Fig. S13c), indicating the ability to sense triboelectric signals quickly at the millisecond level. MTAS measures the two opposite characteristic waveforms by different materials (Fig. S13d). The characteristic waveform of Kapton is negative to positive, whereas that of NBR is positive to negative.

Note S12 Traditional Kitchen Robot

The current kitchen robots can simply stir or heat ingredients prepared by humans. The types of dishes that can be made is generally homogeneous and still requires a deal of human assistance. However, the MTAS kitchen robot with can independently distinguish and select ingredients because of its tactile recognition capabilities.

Note S13 Suitable Storage Areas and Temperatures for Foods in the Kitchen

For example, foods such as bananas and potatoes can be stored at room temperature. A 5 °C refrigerator is the ideal place for keeping foods such as cucumbers and tomatoes fresh. Perishable foods such as meat require about -18 °C to ensure long term storage. The boiled eggs are usually kept in heated containers to ensure that they are delicious when eaten.

Note S14 Programmable Robot Arm Test System and Data Acquisition for Food Recognition in Kitchen Scene

MTAS integrated into the contact bar at the front of the robot arm, enabling autonomous data acquisition. The artificially generated signal interference is substantially excluded, providing a stable test environment, and the recognition accuracy is guaranteed. For more scientific data collection, we strictly standardized environmental parameters and manipulation methods. The laboratory temperature is maintained at 24 °C, the humidity at 19%, and the contact pressure between the robot arm and the food is controlled at 0.1 N. A contact pressure of 0.1 N ensures that the food will not be damaged by the test system or compromised for edible. And the device can perform long-term stable operation under this safety pressure. As a result, the test system is more standardized, excluding the interference of the user or the external environment, and the recognition accuracy is guaranteed.

In addition, the operational approach to data collection is strictly controlled during the triboelectric identification process. Clean the food surface of dirt, moisture and other small molecules of dust before triboelectric recognition. Then, considering the destructive effect of ethanol on the food surface, dust-free paper is used to remove the surface initial charge from the object to further reduce external interference.

In the kitchen, the common types of food are fruits, vegetables, meat, seafood, and spices. Therefore, we selected some representative foods of each category for subsequent multimodal recognition (Fig. S15a).

Note S15 Food Identification Dataset

The dataset for multimodal learning part of the kitchen scene contains 1800 samples (classifying 18 food items), where each food containing 100 samples. Each sample includes a temperature signal and a triboelectric signal. Depending on the size and shape characteristics of foods, 10 or 5 feature points are selected for sampling 10 or 20 times respectively (the distribution of sampling points is shown in Figs. S16-S24). This strategy avoids errors arising from single-point sampling. The entire learning data for triboelectric recognition and temperature recognition of the 18 foods are presented in Figs. S16-S24.

Note S16 Pre-processing of Machine Learning Data

Kitchen: At each sensing period, our sensor can obtain one sample. Due to the different working mechanisms of the temperature sensor and the triboelectric sensor, the temperature transfer is more time-consuming, which means for a complete sampling of data, the temperature signal will be larger than the triboelectric signal. Through data preprocessing, we align the once-sampled temperature waveform and triboelectric waveform with 1226 and 282 sampling points respectively, as inputs of the neural network.

Mars: Unlike the temperature and the triboelectric waveform in kitchen scene, which has different numbers of sampling points, the pressure and the triboelectric waveform have the same numbers of sampling points, which is set to 275. The training settings are almost the same as those presented in kitchen scene. Moreover, the optimizer's learning rate is set to $1e^{-3}$.

Note S17 Unimodal Machine Learning for Food Recognition

Two unimodal learning approaches are also implemented to classify 18 foods. The corresponding training curves and confusion matrixes are shown in Fig. S25. Both temperature-only network and triboelectric-only network is convergent after around 3000 steps update (Fig. S25c-d), due to the same size dataset and similar network structure. The accuracy of temperature-only network and triboelectric-only network are 37.96% and 90.37% (Fig. S25a-b). Temperature-only network reaches a poor prediction result, where only 5 types of food's accuracy are higher than 60%, and triboelectric-only network can classify 16 types of food with an accuracy above 85%.

Note S18 Preparation and Composition of the Mars Micromodel

A substrate with undulating terrain was prepared by 3D printing that mimics the complex topography on Mars. Separate areas of the microscopic model were covered with rocks of basalt, sand of SiO_2, and homemade simulated Martian soil, which are based on reported soil and rock compositions of Mars [S34, S35]. Thereby, the landform information such as mountains, rocks, land, deserts, and dry rivers on Mars are simulated.

Note S19 Exploring Topographic and Geomorphic Features of Micromodels by Mars Test System

When exploring the topographic and geomorphic features, it needs to initiate constant force touch mode first (through the communication feedback of the robot arm and the force control device) to obtain the terrain undulation information of the micromodel. Second, reconstructing a 3D simulation map with Matlab fitting. (Figs. 4d and S26a). According to the topographic reconstruction map, the geomorphic feature points are identified. Then, the position and contact strength of MTAS are adjusted depending on the altitude and topography of the feature points. After that, multifunctional sensing signals (pressure + triboelectric) of micromodel are collected by the programmable movements of the robot arm. Finally, the acquired data are recognized by combining with multimodal learning algorithm. During the test, the laboratory temperature was controlled at 24 °C, the humidity was 19%, and the contact pressure between the robotic arm and the micromodel was 0.1 N. A standardized test environment is built to reduce human interference and ensure the accuracy and reliability of recognition.

Note S20 Mars Identification Dataset

The dataset for multimodal learning part of the Mars scene contains 400 samples and 100 samples for each landforms type. Each sample includes a pressure signal and a triboelectric signal.

Note S21 Feature Waveforms for Landforms Identification

The entire learning data for triboelectric identification and pressure identification of the four landforms are demonstrated in Figs. S27-S28. Among them, the waveforms of labels 3 and 4 are from negative to positive, and the rest of the waveforms are from positive to negative. And different landforms exhibit different signal amplitudes (Fig. 4e).

Note S22 Fine-tuning of the Multimodal Recognition Framework

we proposed task-independent universal training framework, providing robust classification results. By fine-tuning inputs and outputs of network and hyperparameters of training process, it can quickly adapt to new classification tasks in variety of scenes, dramatically simplifies the sophisticated steps of repeatedly building neural networks. As shown in Figs. S30-S31 and Table S2, minor changes are made to accommodate the Mars scene, including the number of neurons for the 1D convolutional layer, the number of convolutional layers, the dimensions of the input data and the training hyperparameters.

Note S23 Training Curves and Confusion Matrixes of Unimodal Learning Approaches for Micromodel Recognition

Figure S26d shows that pressure-only network is updated around 600 steps before becoming convergent, while Fig. S26e indicates that triboelectric-only network is well trained around 400 steps. Figure S26b-c are the confusion matrix for pressure-only network and triboelectric-only network, respectively. The accuracy of pressure-only network and triboelectric-only network are 80.83% and 91.67%, respectively.

Note S24 Waterproof Performance

Due to the great water repellency of PE, ecoflex and acrylic glue, MTAS has an excellent waterproof function. MTAS had no performance impairment in either mode after continuous or reciprocal operation underwater for 4 h (Fig. S32d-g). In addition, further testing of the pressure,

temperature and triboelectric signals after the device was completely submerged for 4 hours, and again, no damage occurred for each sensing function.

Note S25 Characteristic Recognition of MTAS Generated by Contact with Water Interface

When just contacting or leaving the water surface, it produces a sharply rising waveform that reaches a spike and then falls more gently (Fig. S32a-c). This may be the medium change when MTAS passes through the gas-liquid interface, resulting in a sharp vertical rise in the signal, indicating that MTAS can characteristically identify the water signal.

Supplementary Figures and Tables

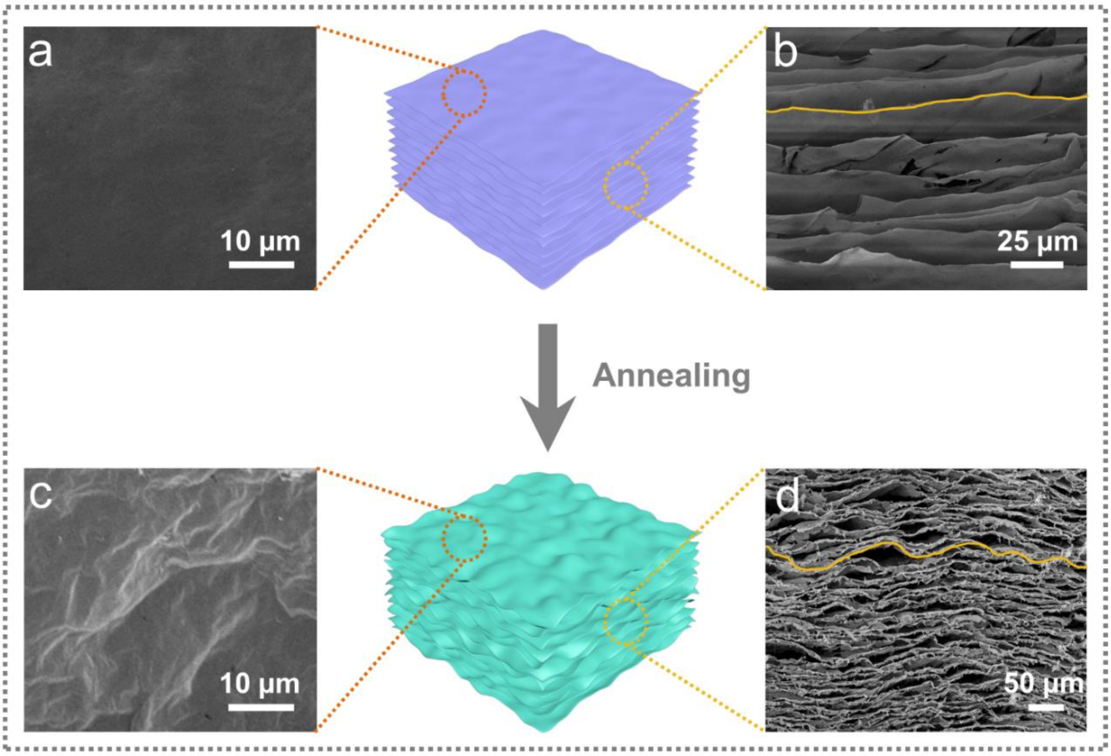

Fig. S1 Microscopic characteristics of MTAS. a) SEM images of vertical the flat GO/CNCs aerogel lamellae orientation. **b**) SEM images along the flat GO/CNCs aerogel lamellae orientation. **c**) SEM images of vertical rGO/CNCs aerogel lamellae orientation. **d**) SEM images along the wavy rGO/CNCs aerogel lamellae orientation

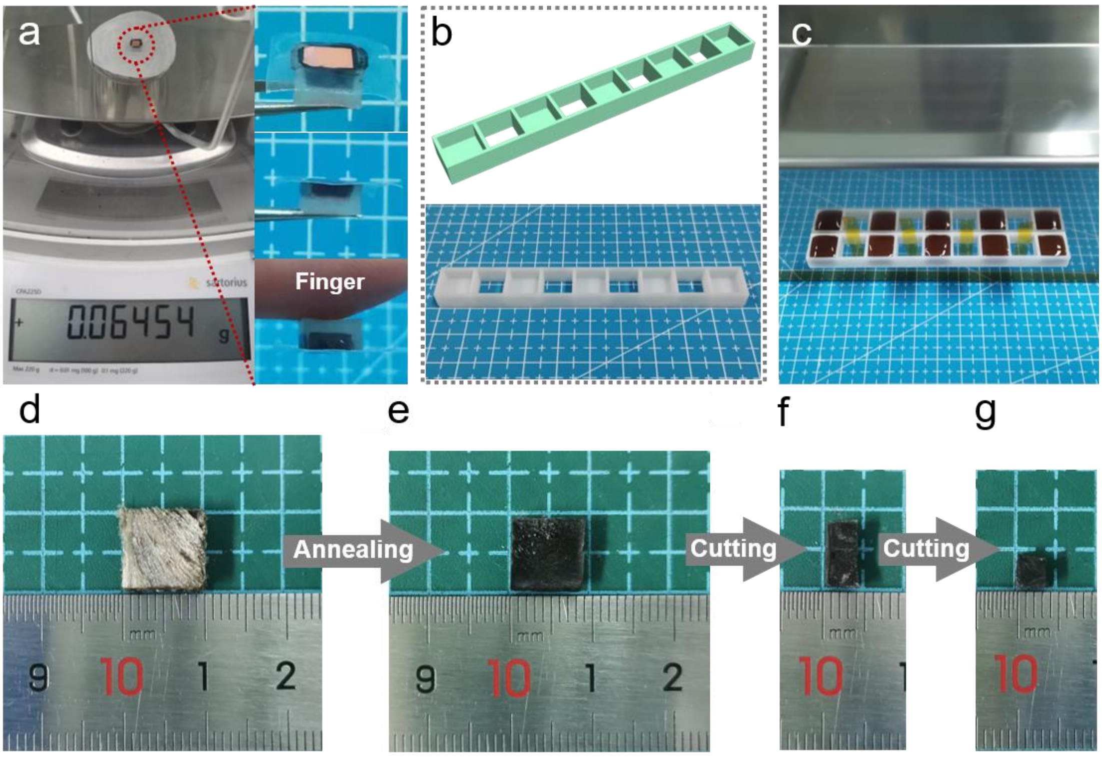

Fig. S2 a) The ultralight characteristics of MTAS and photos from different angles. **b**) The 3D model and photograph of freeze-drying mold. **c**) Photograph of a mold fixed to the side wall of a steel box to directional freezing. **d**) Photo of rGO/CNCs aerogel after freeze-drying. **e**) Photo of rGO/CNCs aerogel after annealing. **f, g**) Photo of rGO/CNCs aerogel after cutting

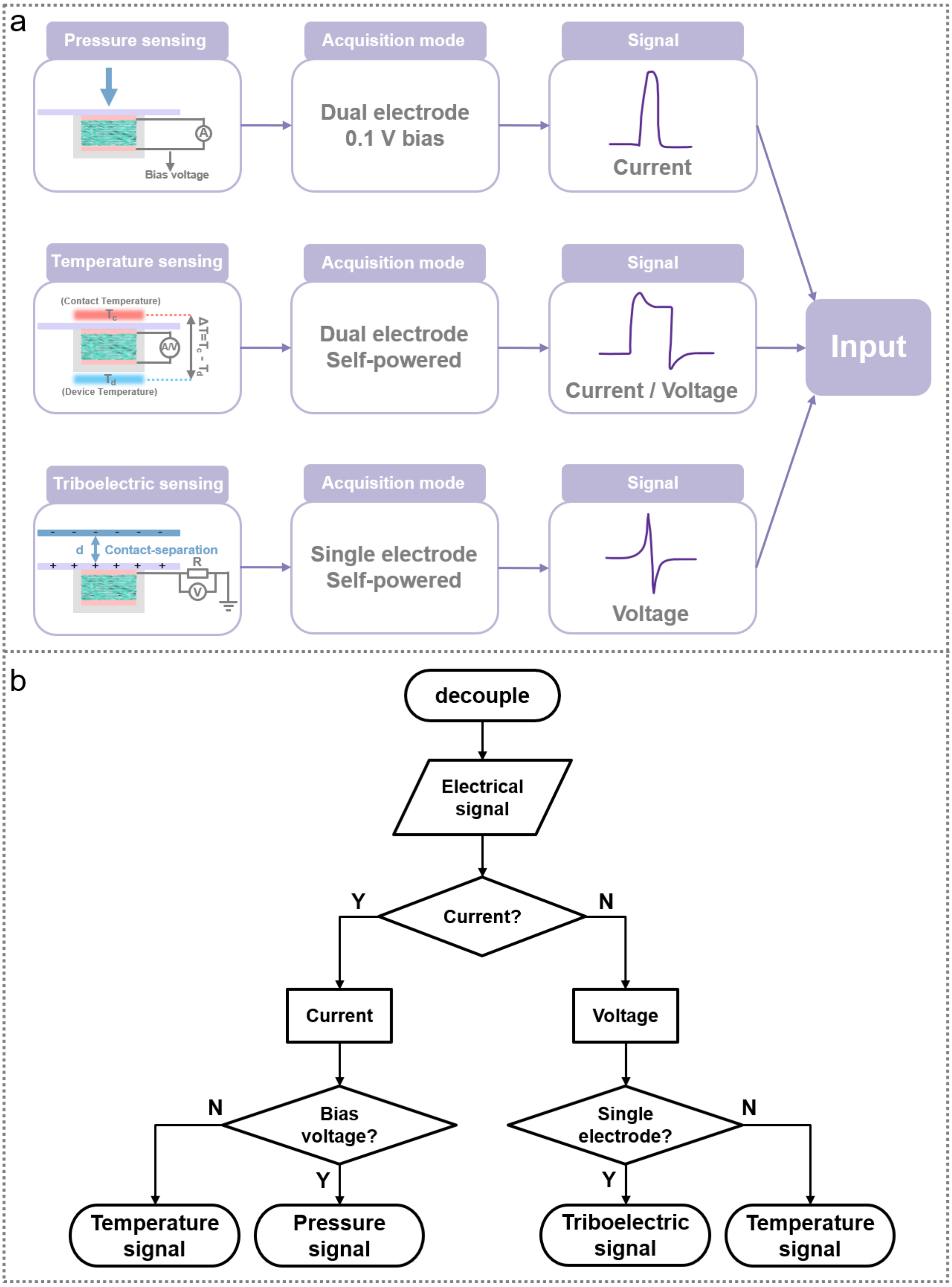

Fig. S3 Acquisition method and decoupling of multifunctional sensing signals. a) Acquisition method of multifunctional sensing signals. **b**) Flow chart of decoupling multifunctional sensing signals

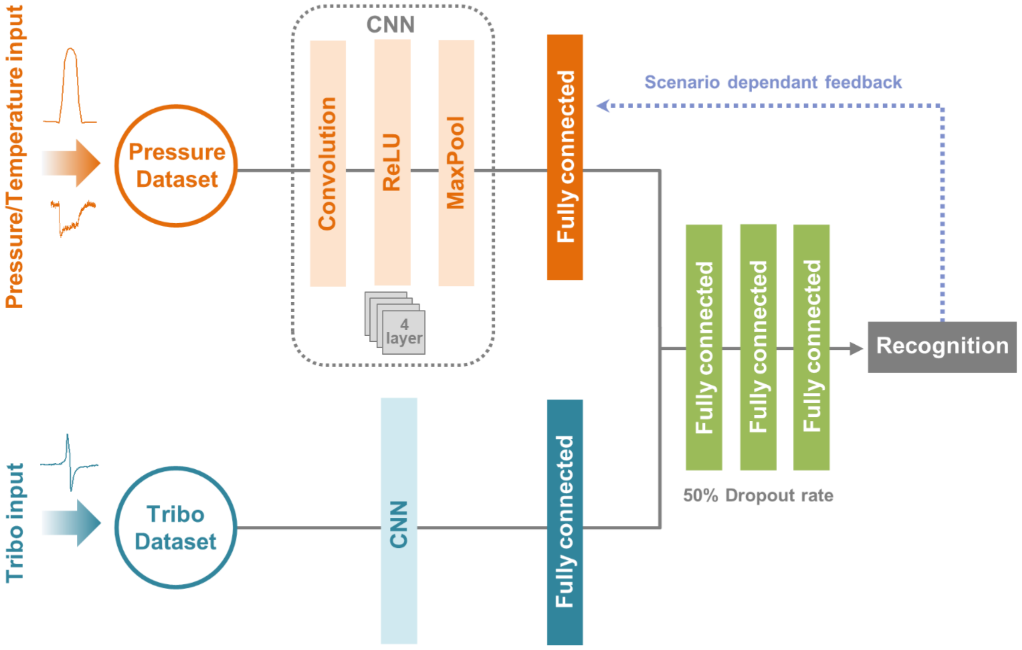

Fig. S4 Multimodal machine learning algorithm architecture based on MTAS haptic system

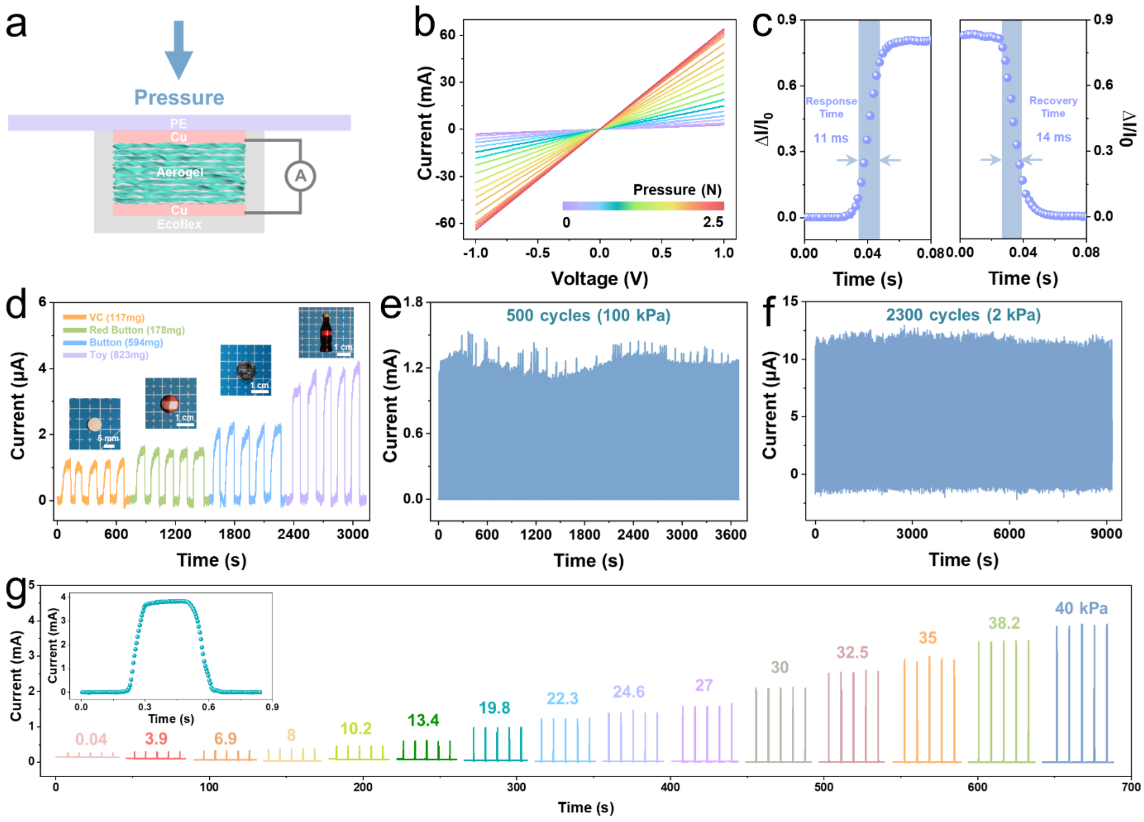

Fig. S5 Performance characterization of MTAS for pressure. a) Schematic of the pressure sensing mode. When the pressure is applied vertically, the elastic MTAS can convert the pressure information into current signals in the external circuit. **b**) The *I-V* curves of MTAS at different pressures from 0.001 to 2.5N (100kPa). **c**) Response time of MTAS pressure sensing. **d**) MTAS detects the output signal of the four ultralight objects. **e**) Stability test 500 cycles at a pressure of 100 kPa. **f**) Stability test 2300 cycles at a pressure of 2 kPa. **g**) Current curves of MTAS at different pressures. Inset: Current response signal for a single pressure cycle

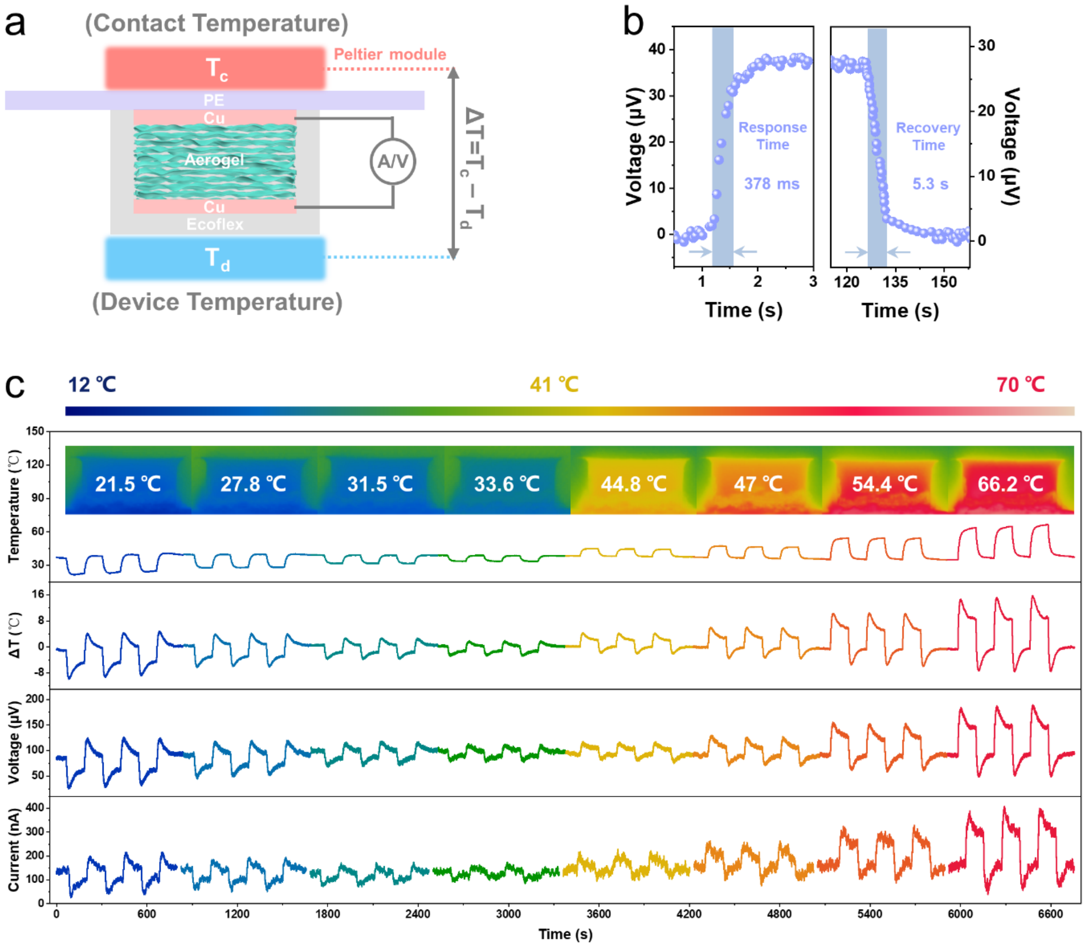

Fig. S6 Performance characterization of MTAS for self-powered temperature sensing. a) Schematic of the temperature sensing mode. **b**) Response time of MTAS temperature sensing. T_c represents the contact temperature (absolute surface temperature) and T_d represents the device temperature. ΔT represents the temperature difference ($\Delta T = T_c - T_d$). **c**) The absolute surface temperature (contact temperature, T_c), temperature difference (ΔT) between the upper and lower surfaces, the voltage and current response of MTAS at different temperatures. Inset: Infrared thermal images at different temperatures, whose colors correspond to the colors of the output signal curve below. MTAS was placed upside down above the Peltier variable temperature module for testing.

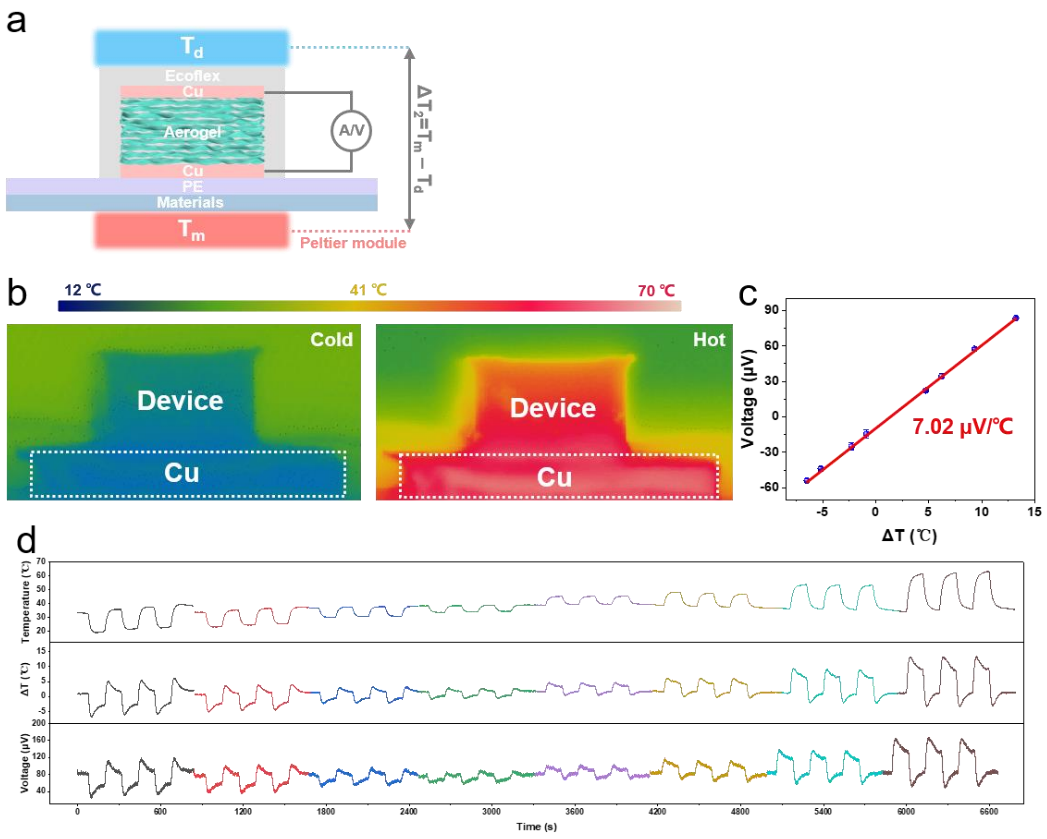

Fig. S7 The heat transfer properties characterization of 11 materials. a) Schematic diagram of the heat transfer performance test. The measurement materials were placed between the Peltier module and inverted MTAS. **b**) Infrared photograph of Cu thermal transfer performance testing. **c**) Voltage temperature sensitivity as influenced by the heat transfer capability of the material. **d**) Curves of temperature, temperature difference and voltage for simultaneous testing at different temperatures

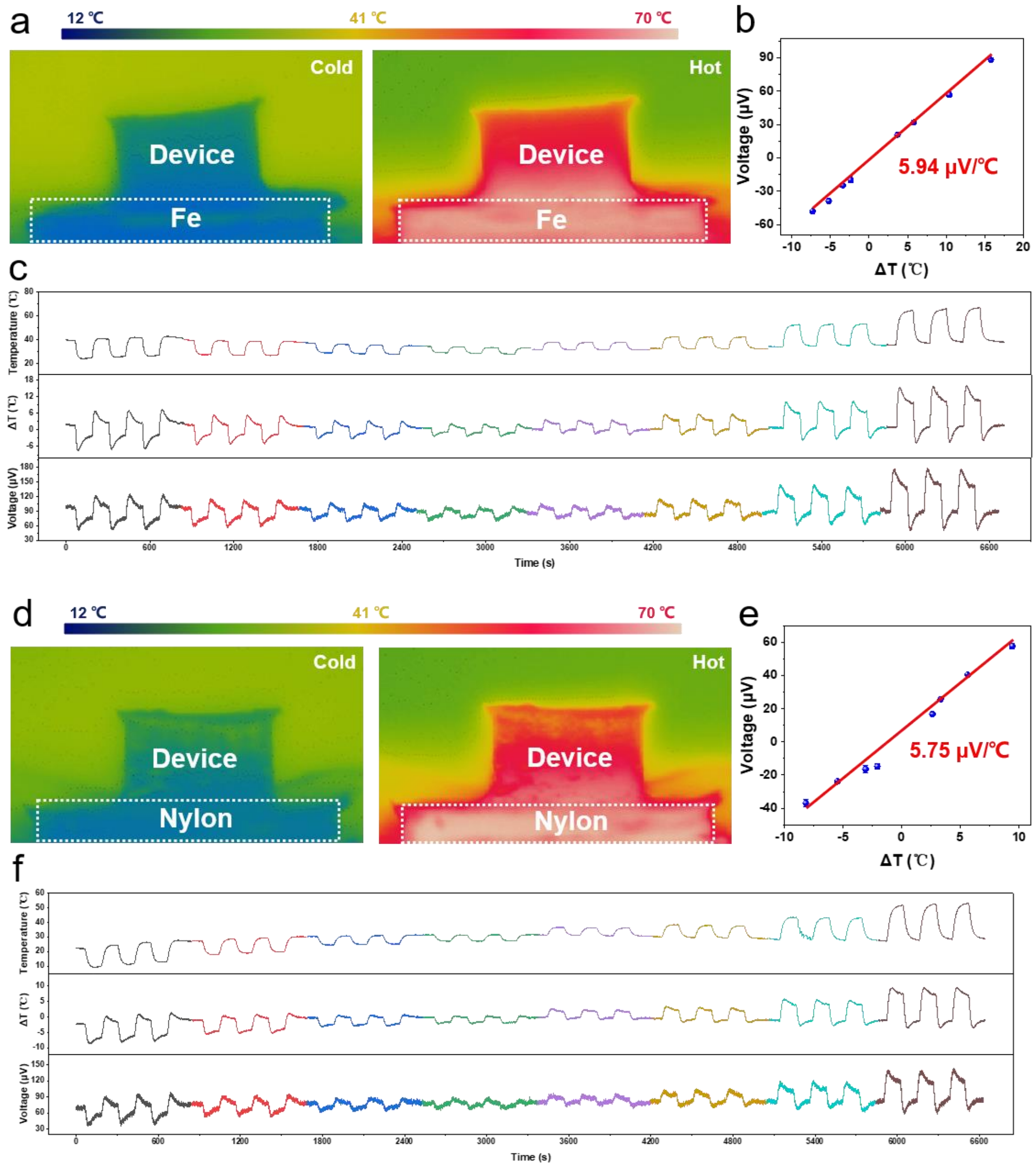

Fig. S8 The heat transfer properties characterization of Fe and Nylon. a) Infrared photograph of Fe thermal transfer performance testing. **b**) Voltage temperature sensitivity as influenced by the heat transfer capability of the material. **c**) Curves of temperature, temperature difference and voltage for simultaneous testing at different temperatures. **d**) Infrared photograph of Nylon thermal transfer performance testing. **e**) Voltage temperature sensitivity as influenced by the heat transfer capability of the material. **f**) Curves of temperature, temperature difference and voltage for simultaneous testing at different temperatures

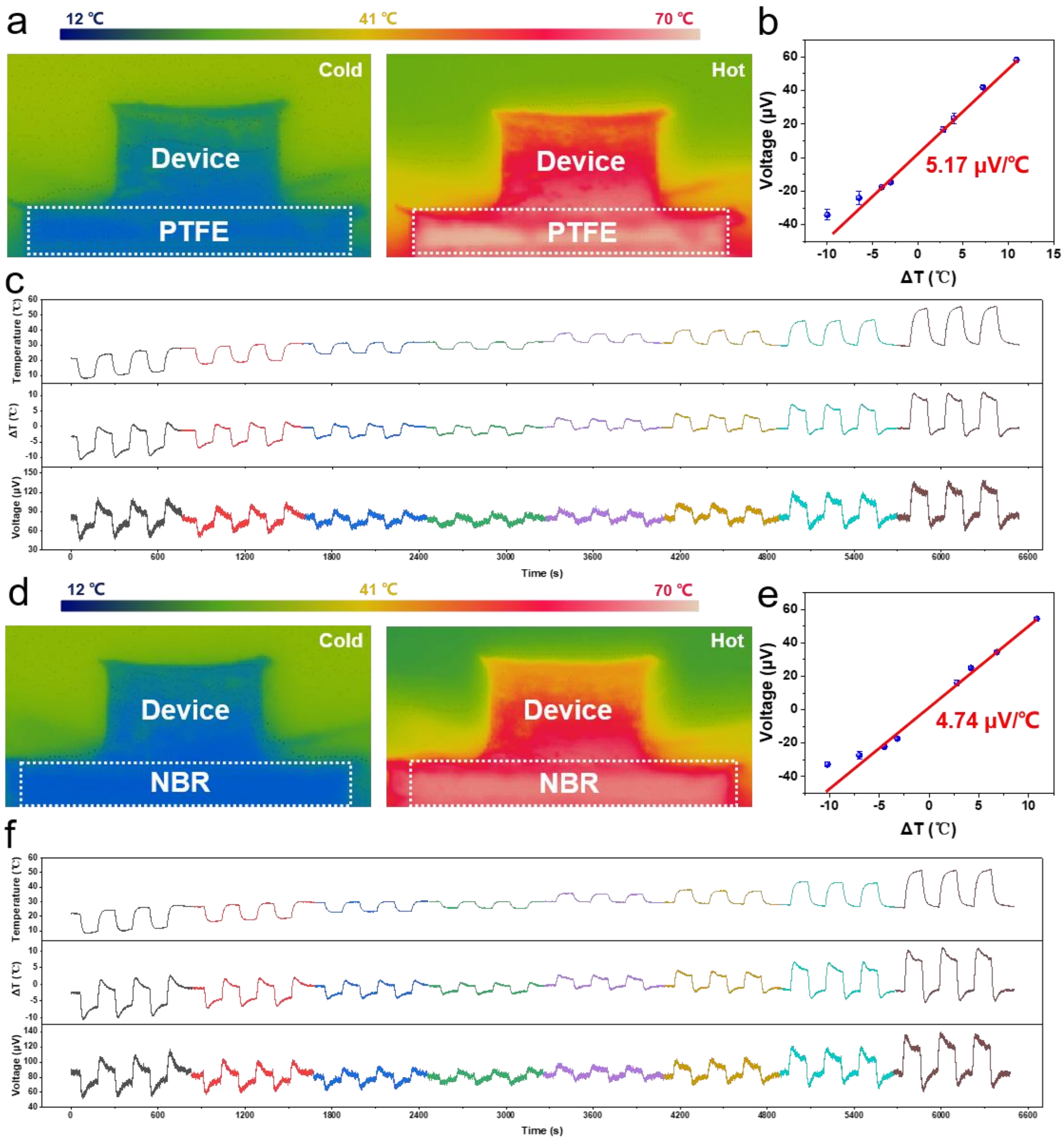

Fig. S9 The heat transfer properties characterization of PTFE and NBR. a) Infrared photograph of PTFE thermal transfer performance testing. **b**) Voltage temperature sensitivity as influenced by the heat transfer capability of the material. **c**) Curves of temperature, temperature difference and voltage for simultaneous testing at different temperatures. **d**) Infrared photograph of NBR thermal transfer performance testing. **e**) Voltage temperature sensitivity as influenced by the heat transfer capability of the material. **f**) Curves of temperature, temperature difference and voltage for simultaneous testing at different temperatures

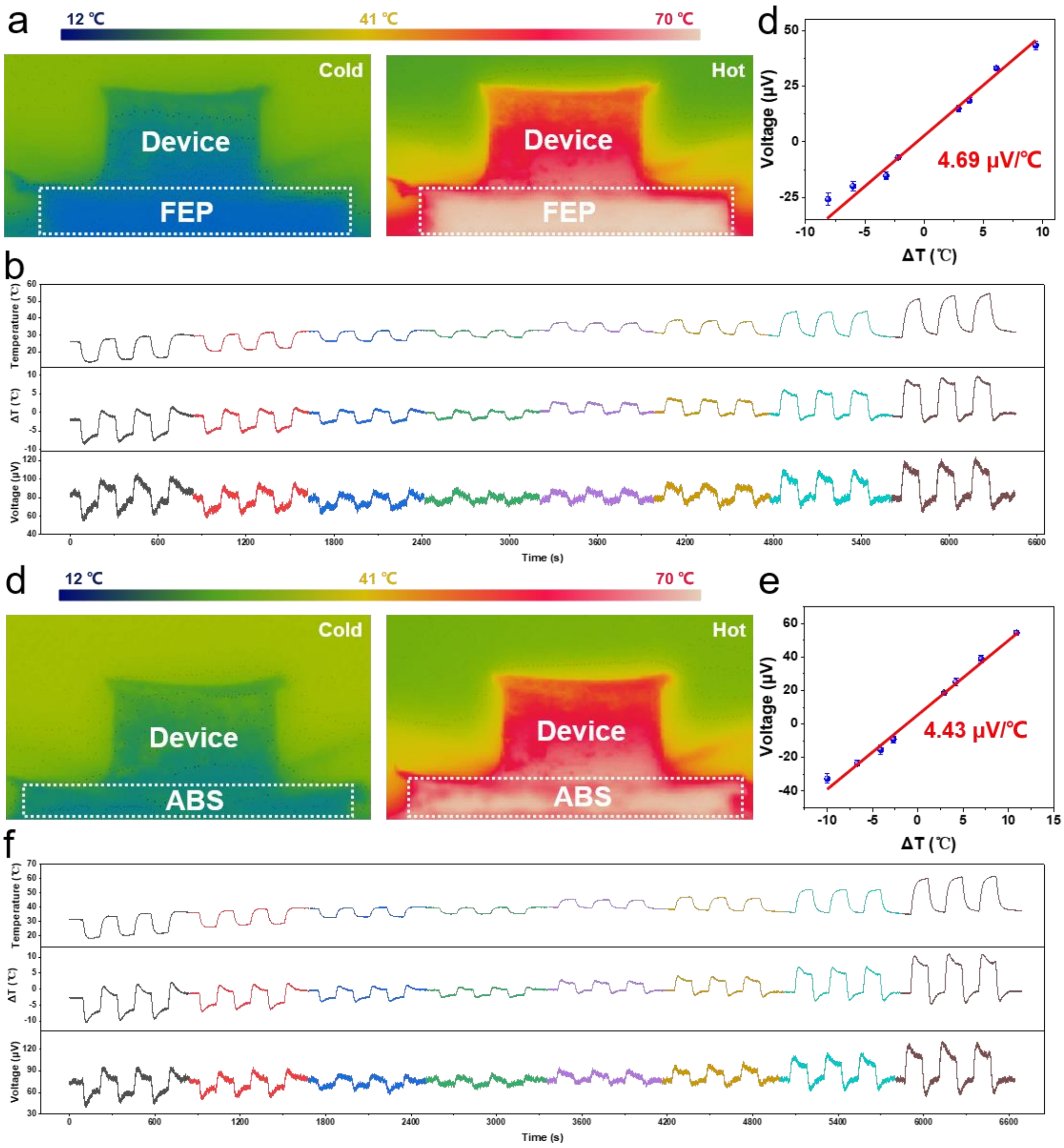

Fig. S10 The heat transfer properties characterization of FEP and ABS. a) Infrared photograph of FEP thermal transfer performance testing. **b**) Voltage temperature sensitivity as influenced by the heat transfer capability of the material. **c**) Curves of temperature, temperature difference and voltage for simultaneous testing at different temperatures. **d**) Infrared photograph of ABS thermal transfer performance testing. **e**) Voltage temperature sensitivity as influenced by the heat transfer capability of the material. **f**) Curves of temperature, temperature difference and voltage for simultaneous testing at different temperatures

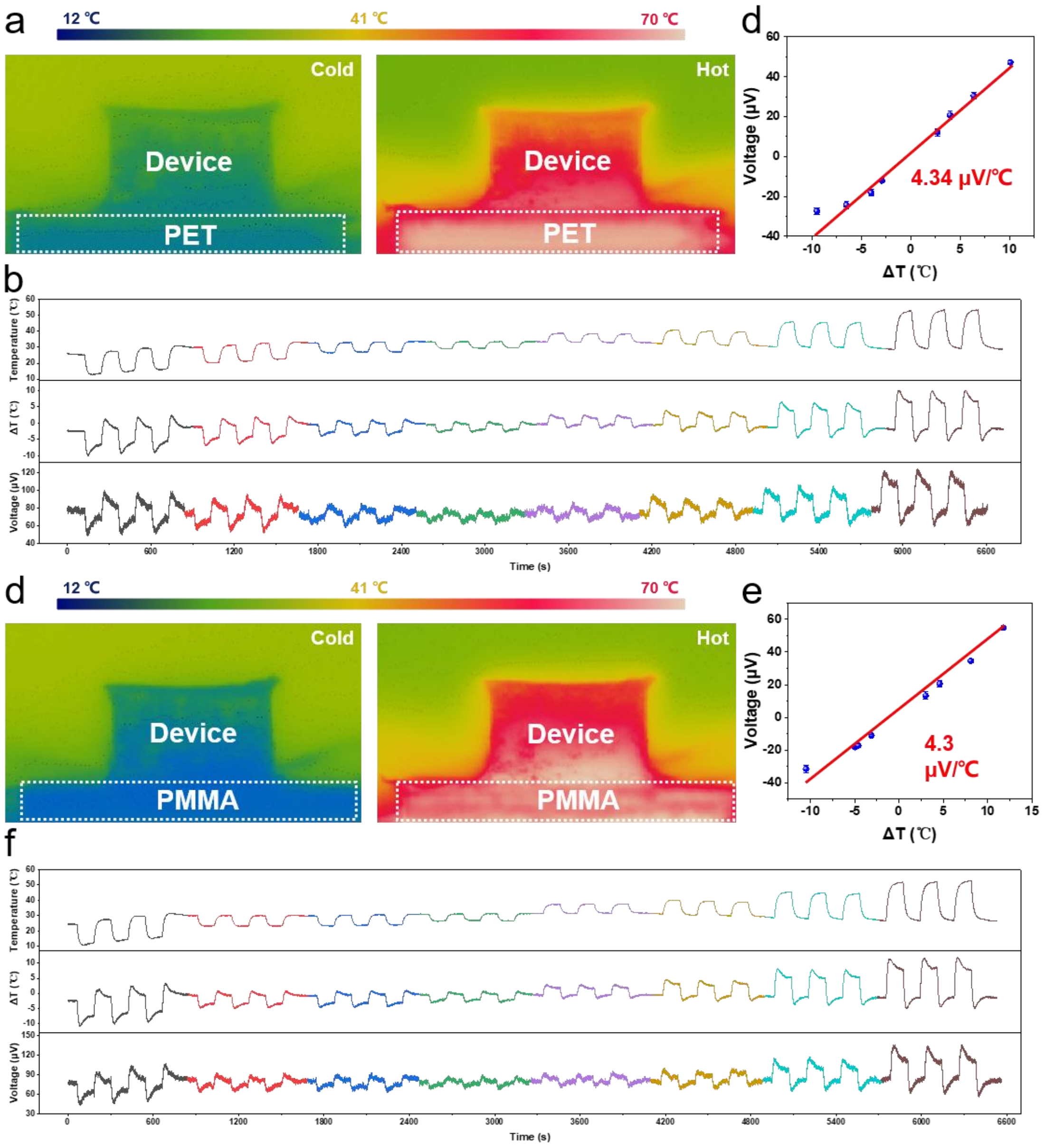

Fig. S11 The heat transfer properties characterization of PET and PMMA. a) Infrared photograph of PET thermal transfer performance testing. **b**) Voltage temperature sensitivity as influenced by the heat transfer capability of the material. **c**) Curves of temperature, temperature difference and voltage for simultaneous testing at different temperatures. **d**) Infrared photograph of PMMA thermal transfer performance testing. **e**) Voltage temperature sensitivity as influenced by the heat transfer capability of the material. **f**) Curves of temperature, temperature difference and voltage for simultaneous testing at different temperatures

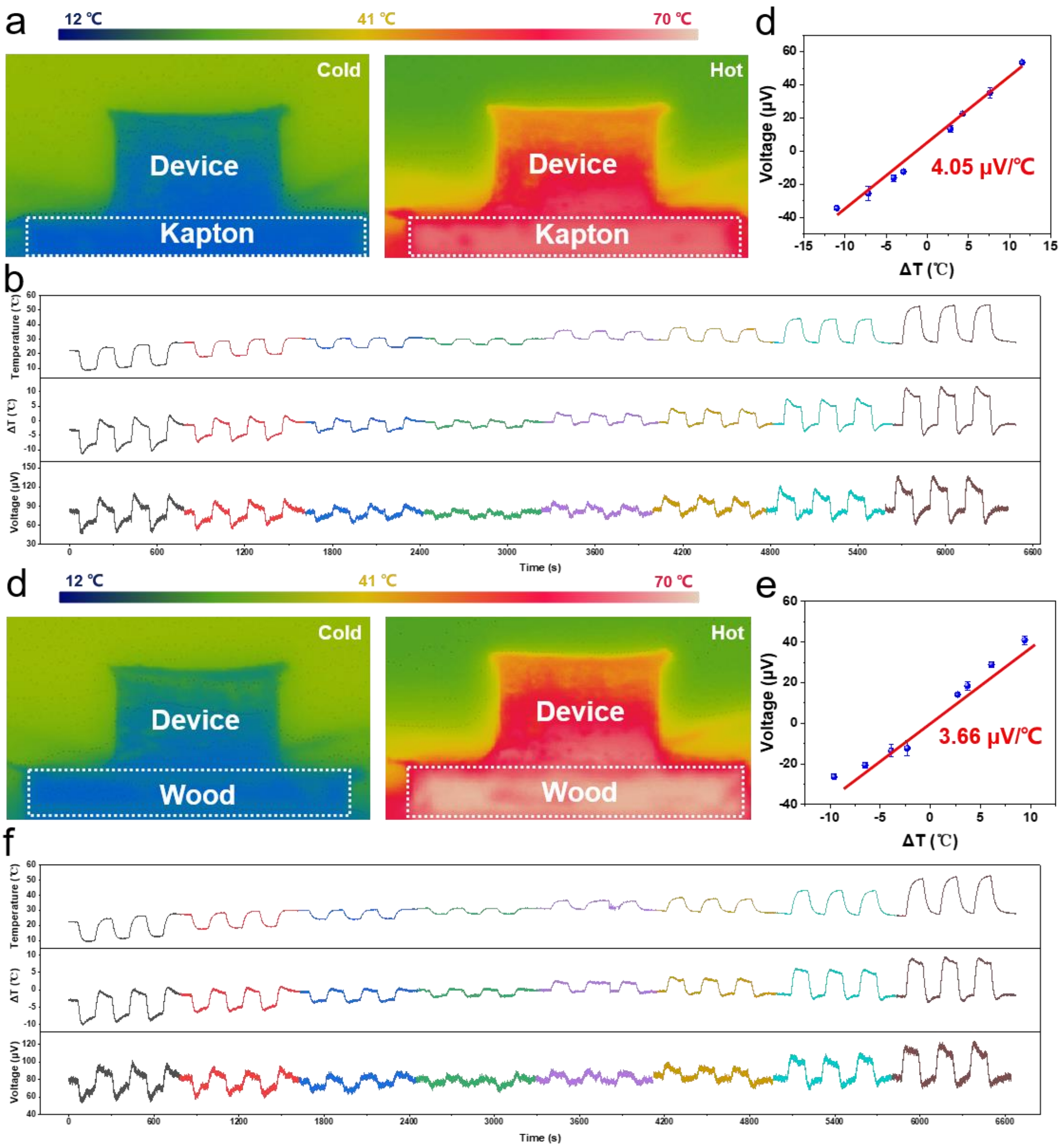

Fig. S12 The heat transfer properties characterization of Kapton and Wood. a) Infrared photograph of Kapton thermal transfer performance testing. **b**) Voltage temperature sensitivity as influenced by the heat transfer capability of the material. **c**) Curves of temperature, temperature difference and voltage for simultaneous testing at different temperatures. **d**) Infrared photograph of Wood thermal transfer performance testing. **e**) Voltage temperature sensitivity as influenced by the heat transfer capability of the material. **f**) Curves of temperature, temperature difference and voltage for simultaneous testing at different temperatures

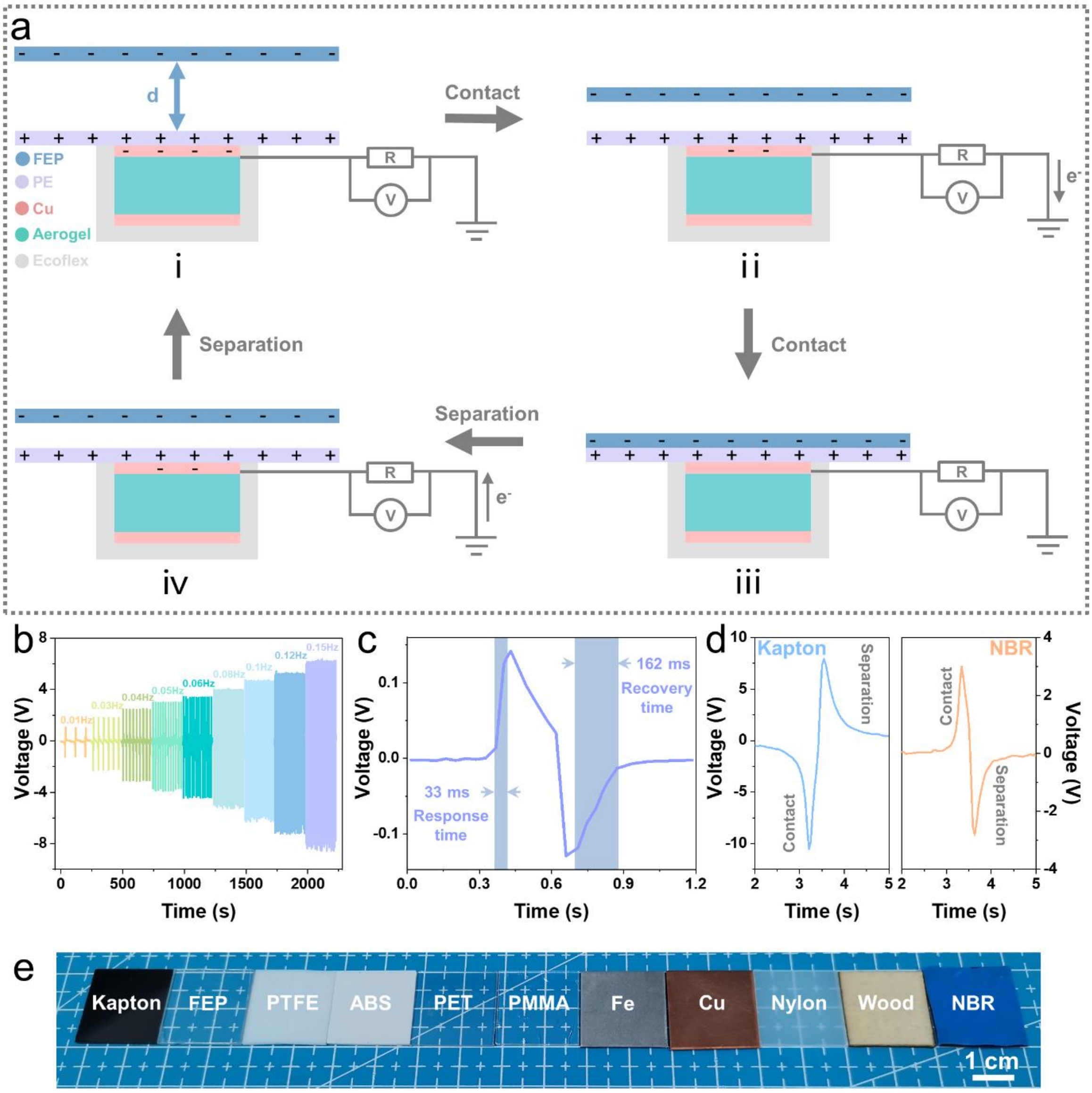

Fig. S13 Characterization of MTAS for the fundamental triboelectric features. a) Working mechanism of triboelectric sensing. **b**) Output voltage at different contact separation frequencies. **c**) Response time of MTAS triboelectric sensing. **d**) Typical characteristic waveforms of two opposite triboelectric signals. **e**) Photographs of 11 materials with the same size (20mm×20mm×1mm)

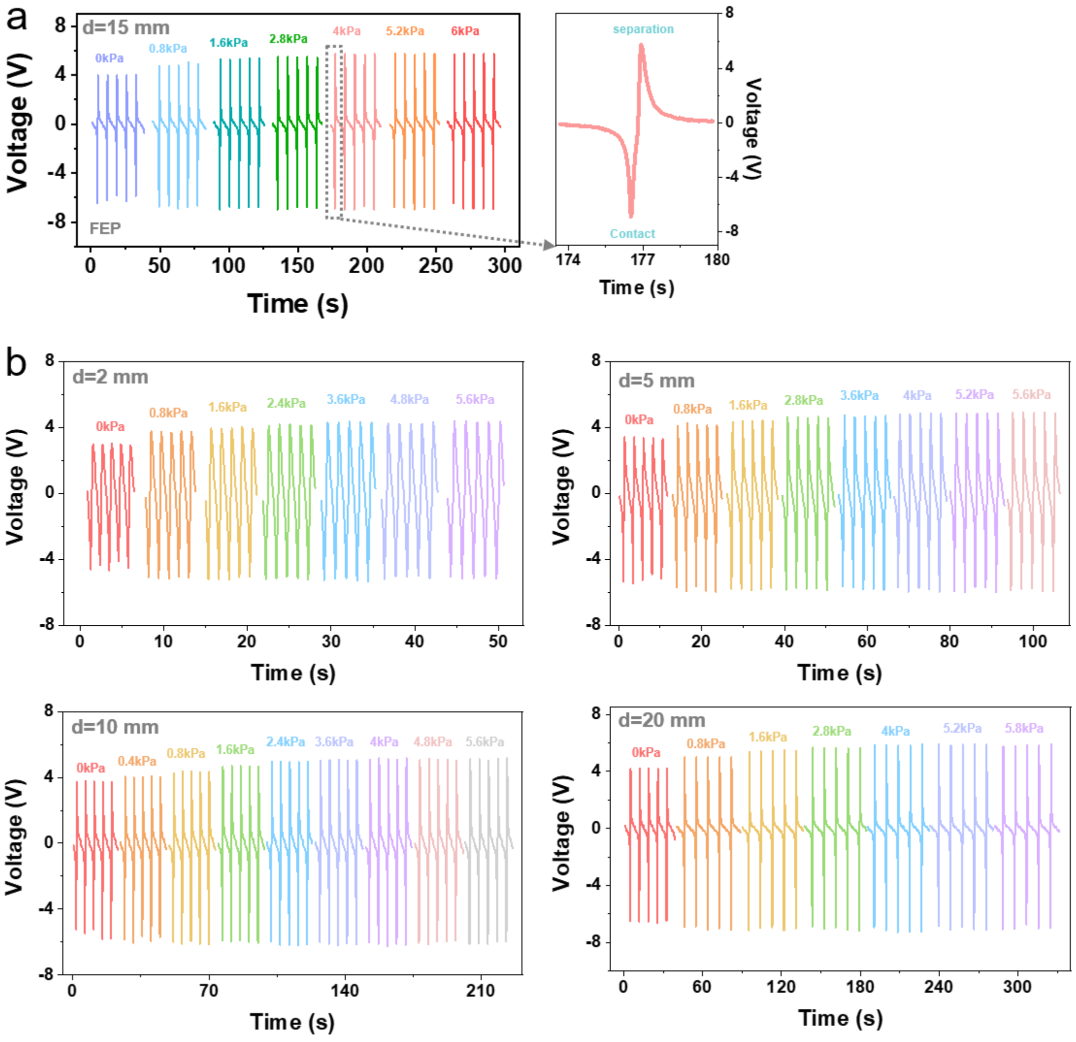

Fig. S14 Effect of pressure on triboelectric signals at different separation distances (d=2 mm, 5 mm, 10 mm, 15 mm and 20 mm). a) The triboelectric sensing signals for different contact pressures at the d of 15 mm. Right: amplification of the voltage signal for a single cycle at 4 kPa contact pressure. The magnification on the right shows the triboelectric signal generated by one cycle of contact-separation motion, obtaining a waveform from negative to positive due to the stronger electronegative of FEP than PE. **b**) The effect of pressure on the output signal at other d

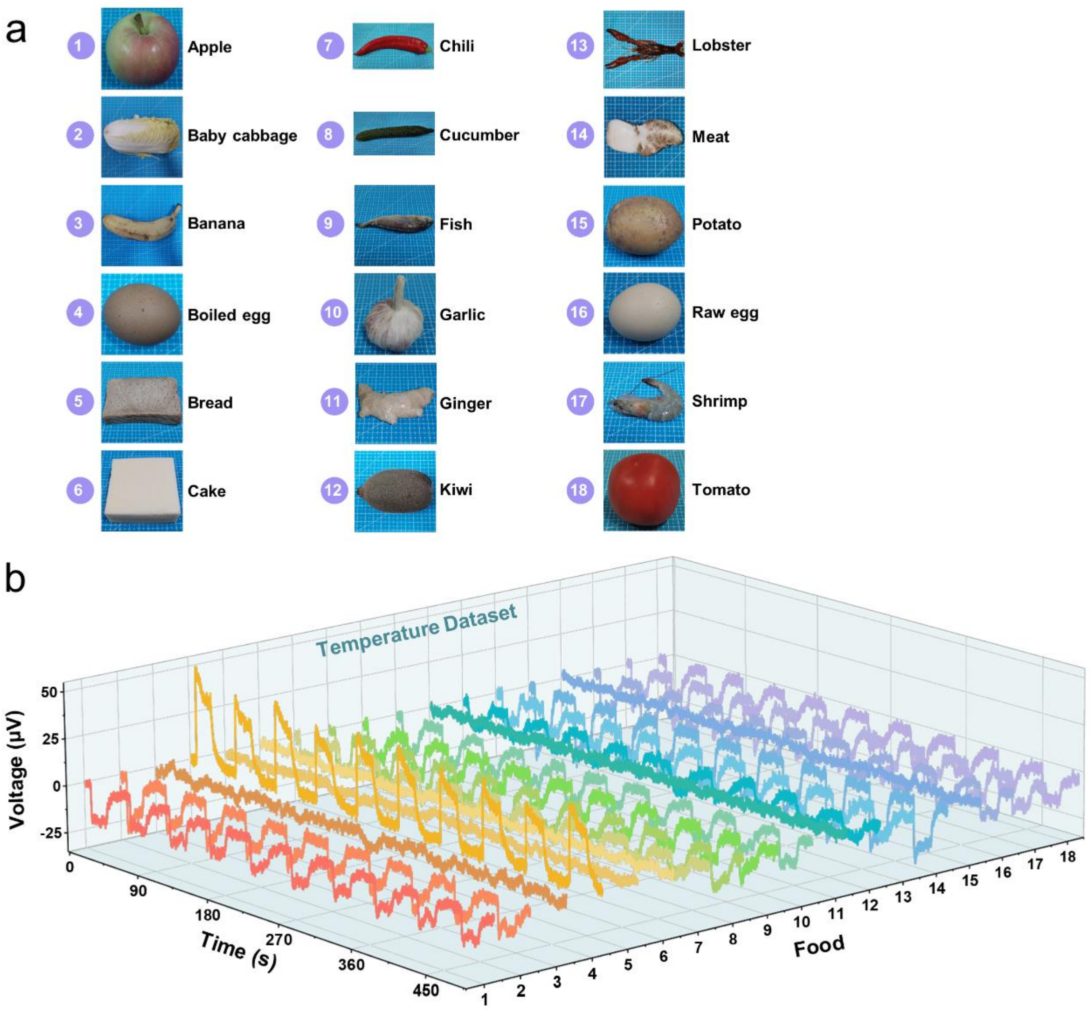

Fig. S15 a) Photographs of 18 kinds of food and their corresponding labels. **b**) 10 cycles of temperature recognition dataset for 18 species of food

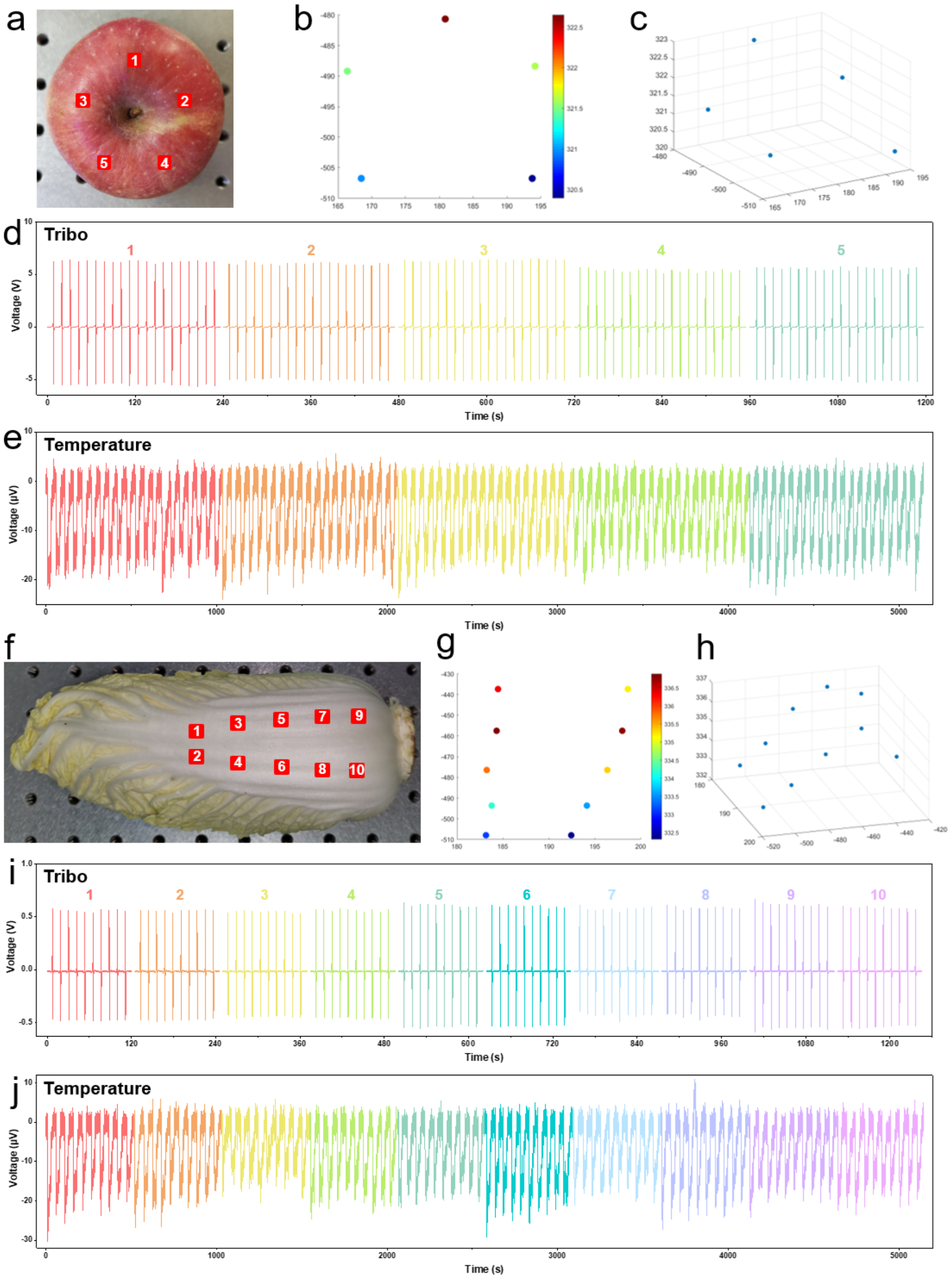

Fig. S16 Machine learning raw data. a) A photograph of feature points distribution on an apple. **b**) Planar simulation of the coordinate distribution of feature points. **c**) 3D simulation of the coordinate distribution of feature points. **d**) Triboelectric recognition signal of apple. **e**) Temperature recognition signal of apple. **f**) A photograph of feature points distribution on a baby cabbage. **g**) Planar simulation of the coordinate distribution of feature points. **h**) 3D simulation of the coordinate distribution of feature points. **i**) Triboelectric recognition signal of baby cabbage. **j**) Temperature recognition signal of baby cabbage

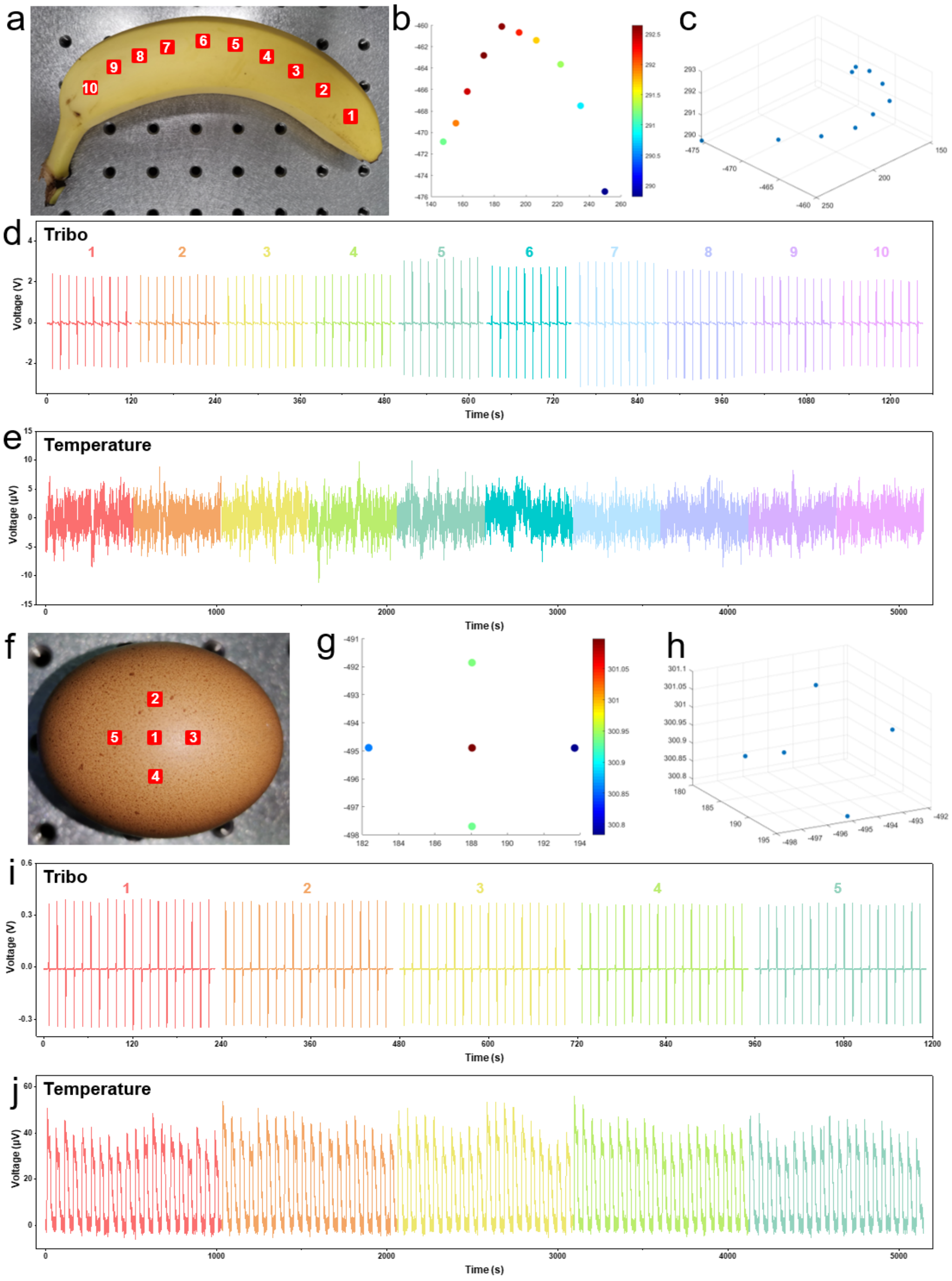

Fig. S17 Machine learning raw data. a) A photograph of feature points distribution on a banana. **b**) Planar simulation of the coordinate distribution of feature points. **c**) 3D simulation of the coordinate distribution of feature points. **d**) Triboelectric recognition signal of banana. **e**) Temperature recognition signal of banana. **f**) A photograph of feature points distribution on a boiled egg. **g**) Planar simulation of the coordinate distribution of feature points. **h**) 3D simulation of the coordinate distribution of feature points. **i**) Triboelectric recognition signal of boiled egg. **j**) Temperature recognition signal of boiled egg

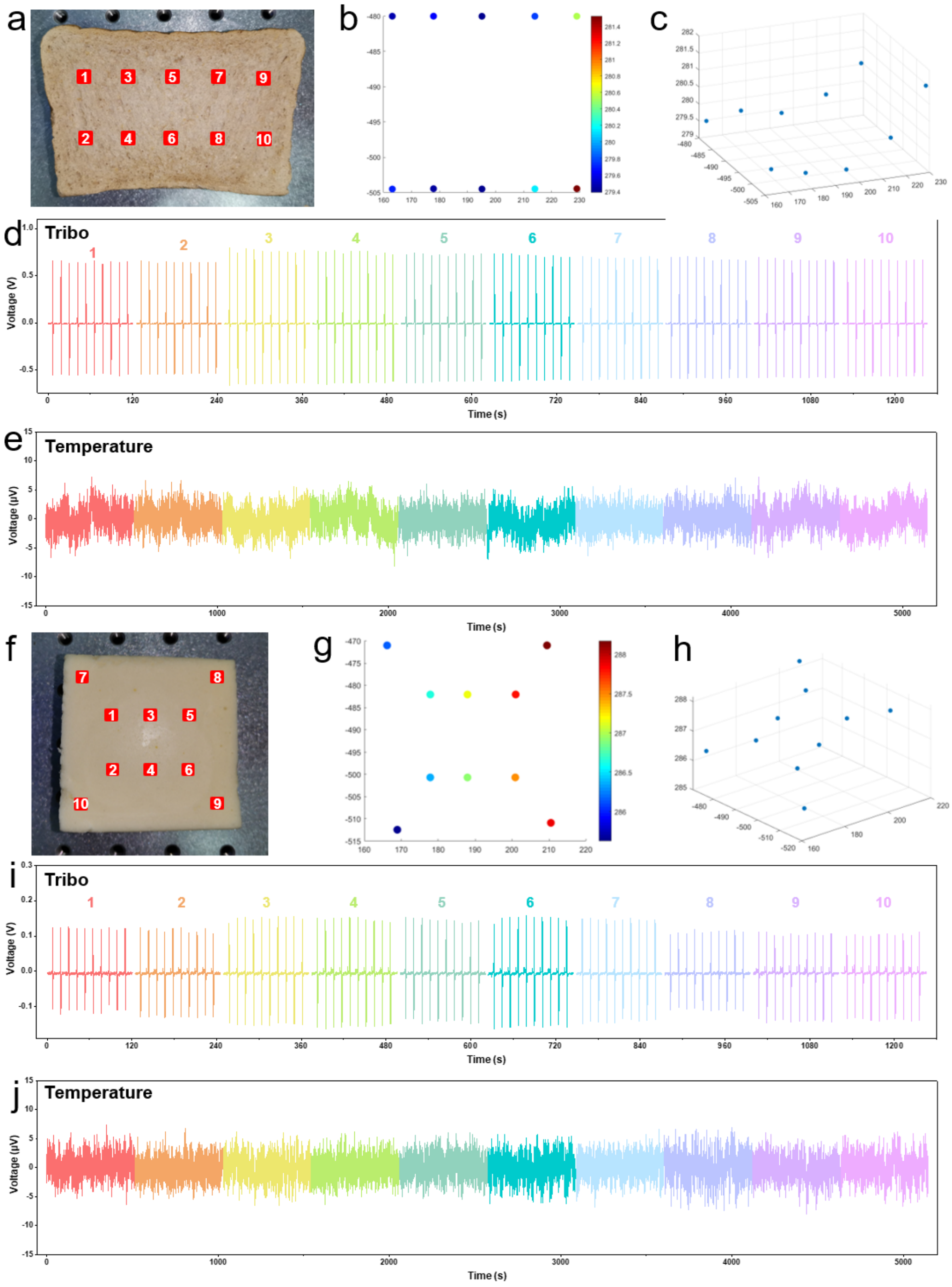

Fig. S18 Machine learning raw data. a) A photograph of feature points distribution on a bread. b) Planar simulation of the coordinate distribution of feature points. **c**) 3D simulation of the coordinate distribution of feature points. **d**) Triboelectric recognition signal of bread. **e**) Temperature recognition signal of bread. **f**) A photograph of feature points distribution on a cake. **g**) Planar simulation of the coordinate distribution of feature points. **h**) 3D simulation of the coordinate distribution of feature points. i) Triboelectric recognition signal of cake. **j**) Temperature recognition signal of cake

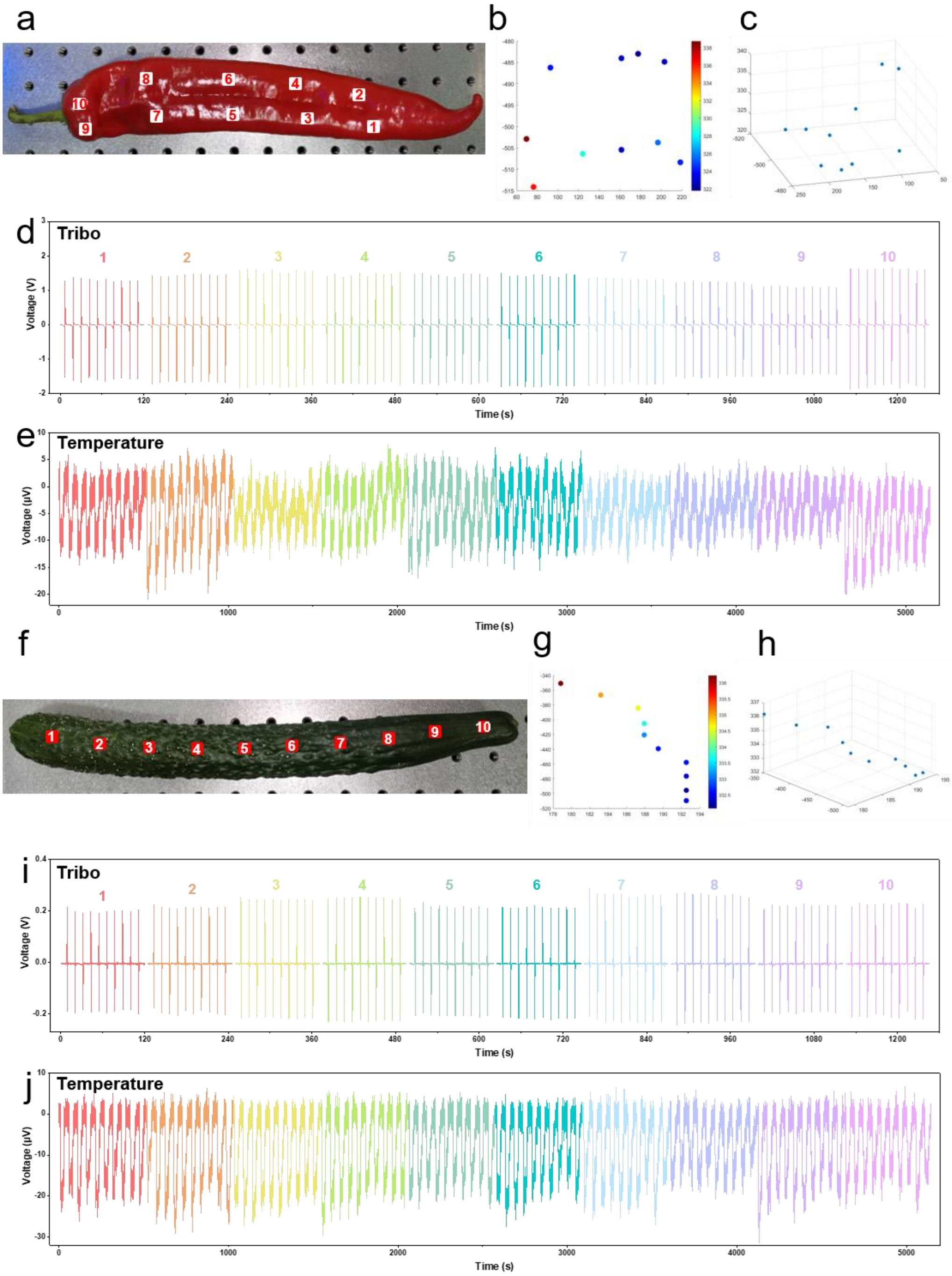

Fig. S19 Machine learning raw data. a) A photograph of feature points distribution on a chili. **b**) Planar simulation of the coordinate distribution of feature points. **c**) 3D simulation of the coordinate distribution of feature points. **d**) Triboelectric recognition signal of chili. **e**) Temperature recognition signal of chili. **f**) A photograph of feature points distribution on a cucumber. **g**) Planar simulation of the coordinate distribution of feature points. **h**) 3D simulation of the coordinate distribution of feature points. **i**) Triboelectric recognition signal of cucumber. **j**) Temperature recognition signal of cucumber

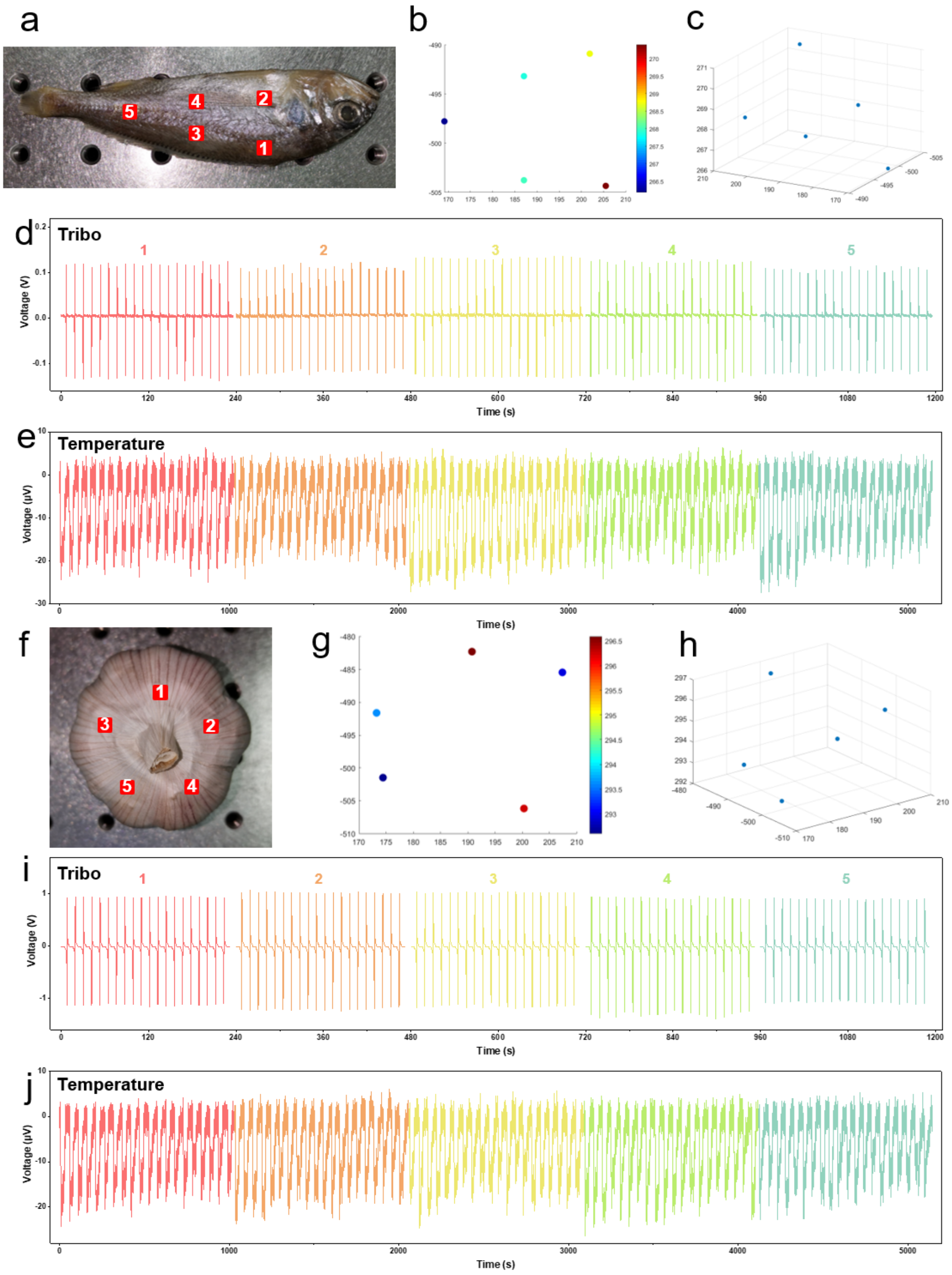

Fig. S20 Machine learning raw data. a) A photograph of feature points distribution on a fish. **b**) Planar simulation of the coordinate distribution of feature points. **c**) 3D simulation of the coordinate distribution of feature points. **d**) Triboelectric recognition signal of fish. **e**) Temperature recognition signal of fish. **f**) A photograph of feature points distribution on a garlic. **g**) Planar simulation of the coordinate distribution of feature points. **h**) 3D simulation of the coordinate distribution of feature points. **i**) Triboelectric recognition signal of garlic. **j**) Temperature recognition signal of garlic

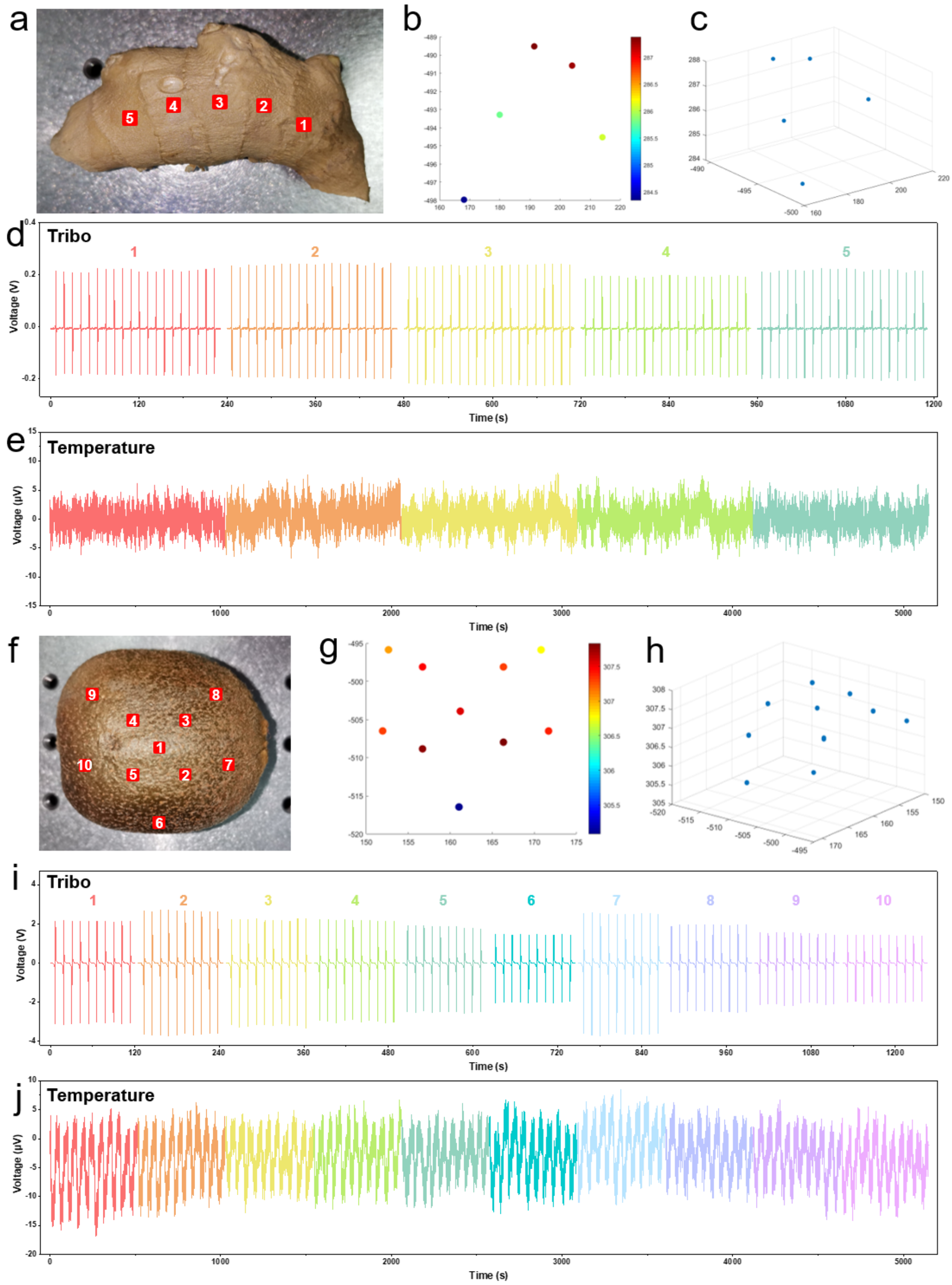

Fig. S21 Machine learning raw data. a) A photograph of feature points distribution on a ginger. **b**) Planar simulation of the coordinate distribution of feature points. **c**) 3D simulation of the coordinate distribution of feature points. **d**) Triboelectric recognition signal of ginger. **e**) Temperature recognition signal of ginger. **f**) A photograph of feature points distribution on a kiwi. **g**) Planar simulation of the coordinate distribution of feature points. **h**) 3D simulation of the coordinate distribution of feature points. **i**) Triboelectric recognition signal of kiwi. **j**) Temperature recognition signal of kiwi

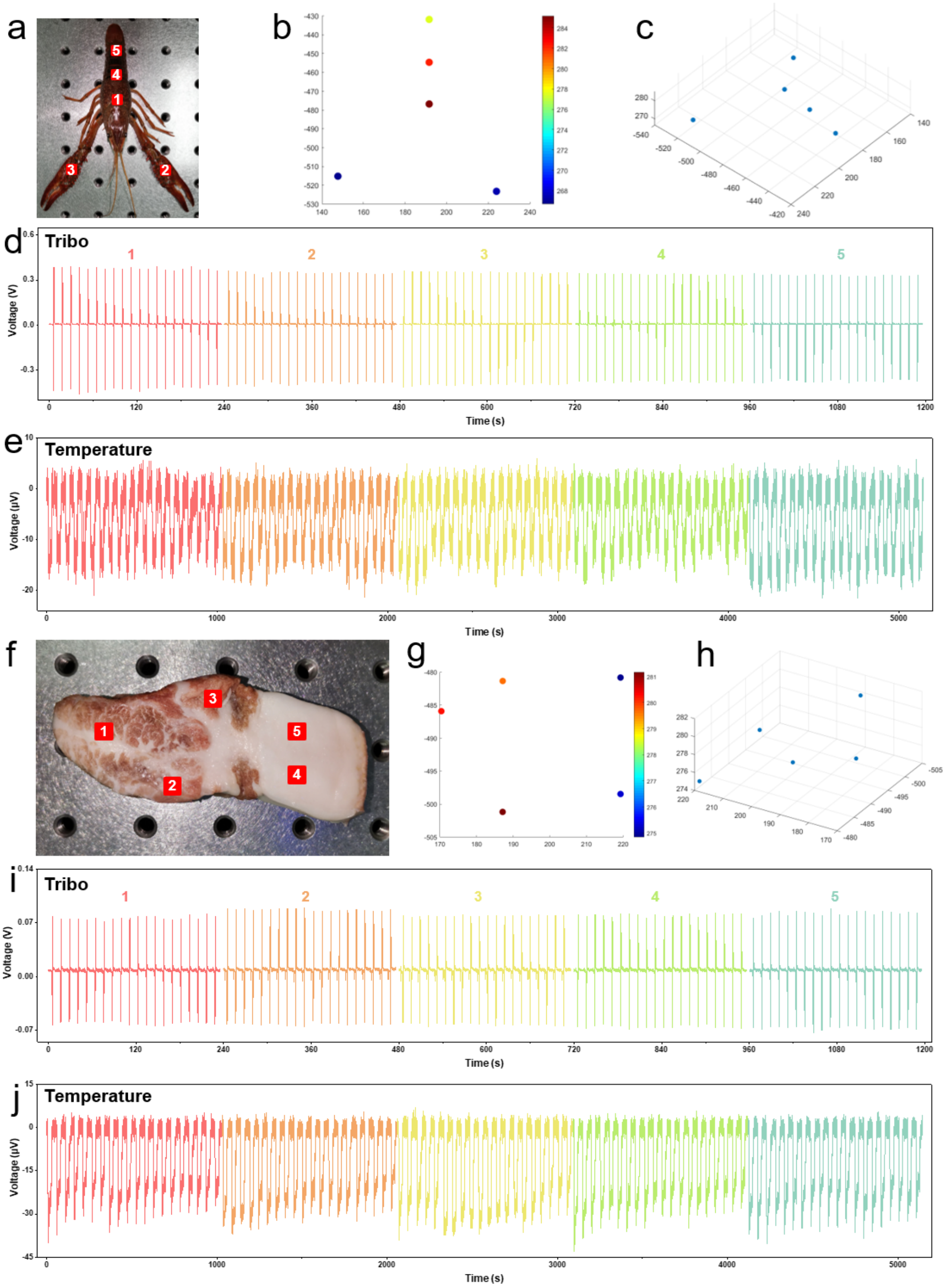

Fig. S22 Machine learning raw data. a) A photograph of feature points distribution on a lobster. b) Planar simulation of the coordinate distribution of feature points. **c**) 3D simulation of the coordinate distribution of feature points. **d**) Triboelectric recognition signal of lobster. **e**) Temperature recognition signal of lobster. **f**) A photograph of feature points distribution on a meat. **g**) Planar simulation of the coordinate distribution of feature points. **h**) 3D simulation of the coordinate distribution of feature points. **i**) Triboelectric recognition signal of meat. j) Temperature recognition signal of meat

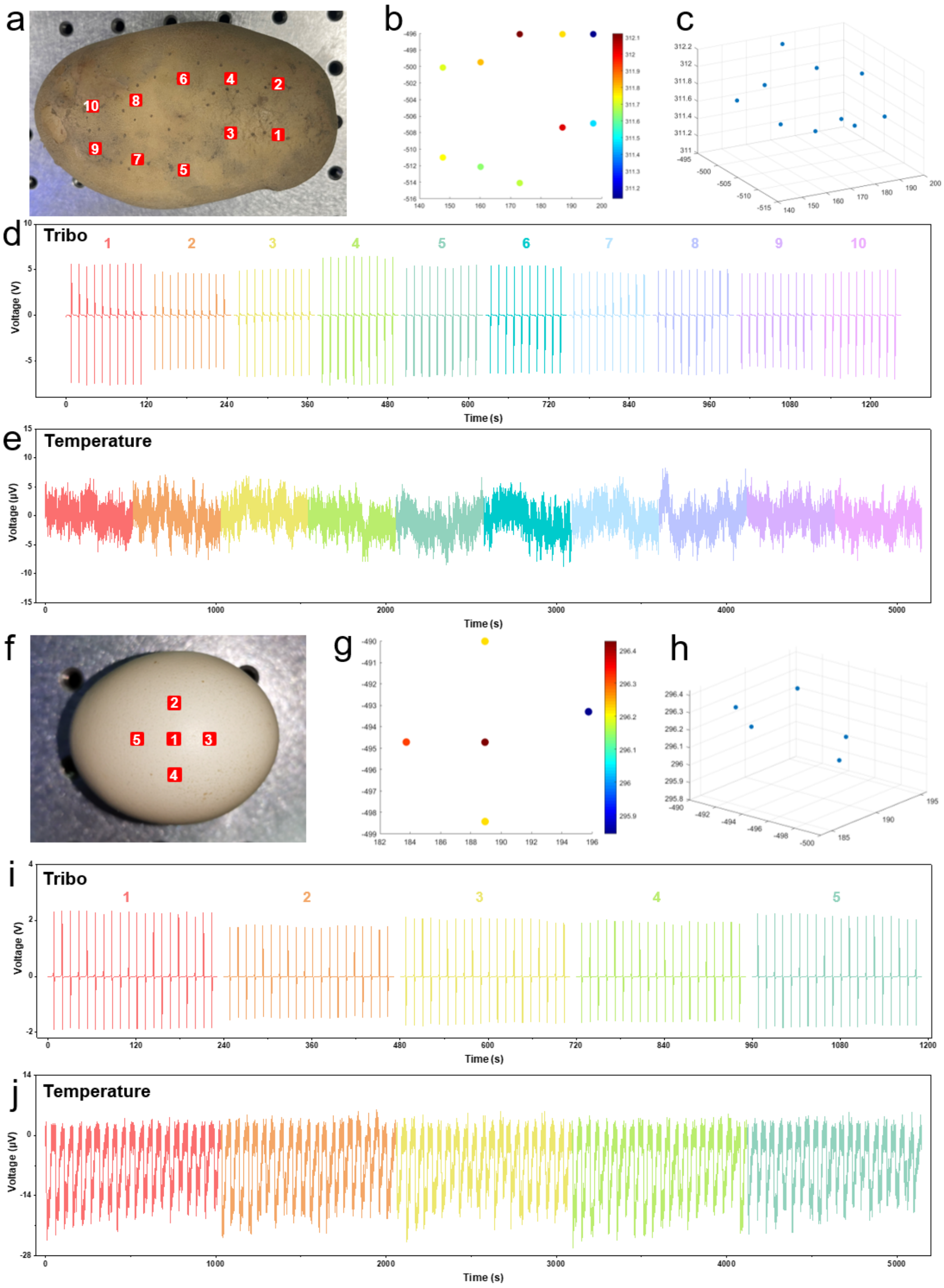

Fig. S23 Machine learning raw data. a) A photograph of feature points distribution on a potato. **b**) Planar simulation of the coordinate distribution of feature points. **c**) 3D simulation of the coordinate distribution of feature points. **d**) Triboelectric recognition signal of potato. **e**) Temperature recognition signal of potato. **f**) A photograph of feature points distribution on a raw egg. g) Planar simulation of the coordinate distribution of feature points. **h**) 3D simulation of the coordinate distribution of feature points. **i**) Triboelectric recognition signal of raw egg. **j**) Temperature recognition signal of raw egg

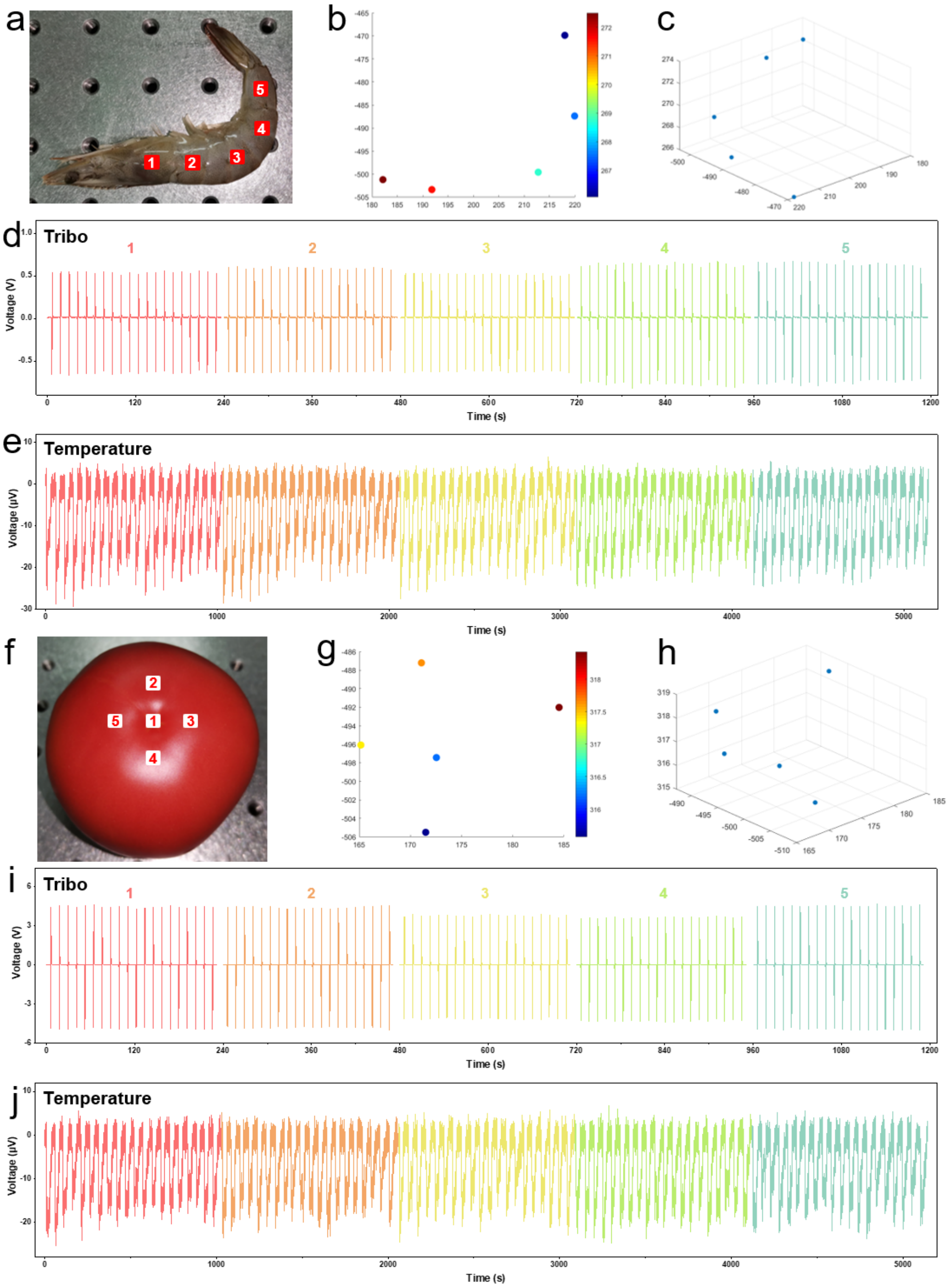

Fig. S24 Machine learning raw data. a) A photograph of feature points distribution on a shrimp. **b**) Planar simulation of the coordinate distribution of feature points. **c**) 3D simulation of the coordinate distribution of feature points. **d**) Triboelectric recognition signal of shrimp. **e**) Temperature recognition signal of shrimp. **f**) A photograph of feature points distribution on a tomato. **g**) Planar simulation of the coordinate distribution of feature points. **h**) 3D simulation of the coordinate distribution of feature points. **i**) Triboelectric recognition signal of tomato. **j**) Temperature recognition signal of tomato

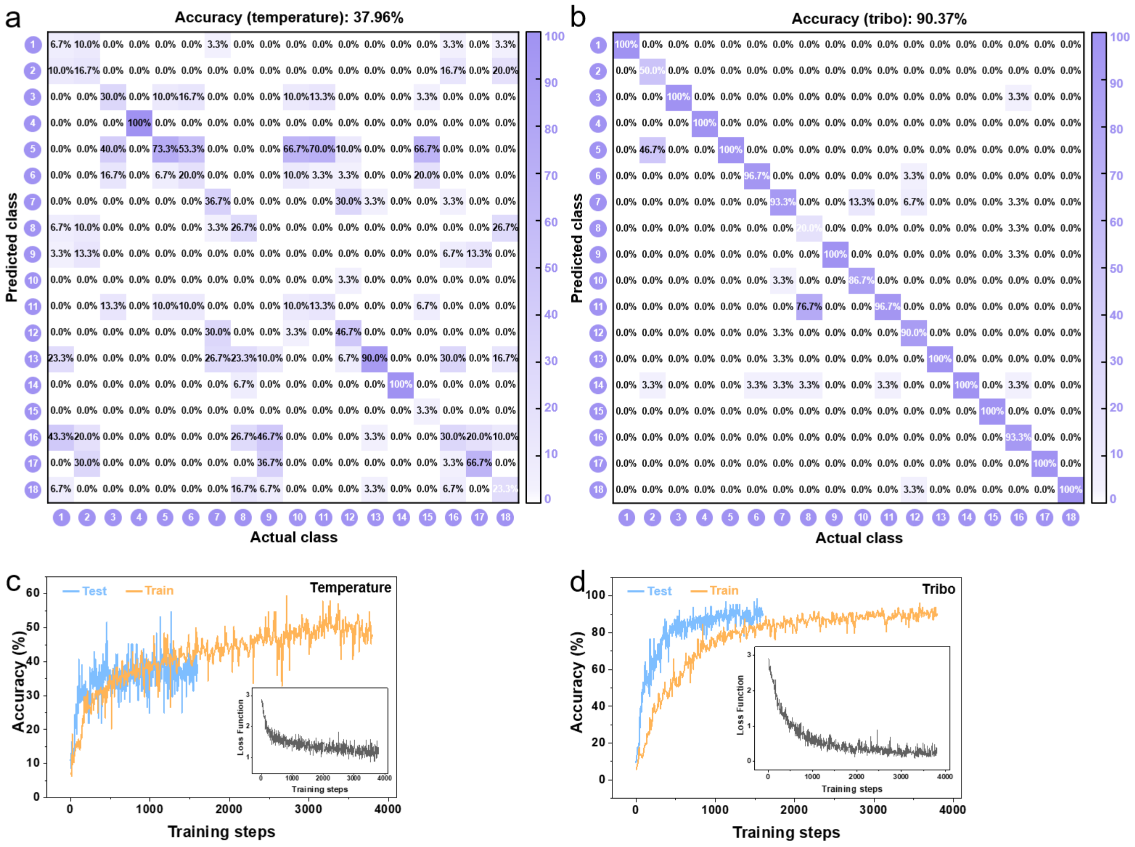

Fig. S25 Unimodal recognition results for kitchen scenes. a) Confusion matrix for temperature identification of food. **b**) Confusion matrix for triboelectric identification of food. **c**) Curve of temperature recognition accuracy with training steps. Inset: Curve of loss functional with training steps. **d**) Curve of triboelectric recognition accuracy with training steps. Inset: Curve of loss functional with training steps

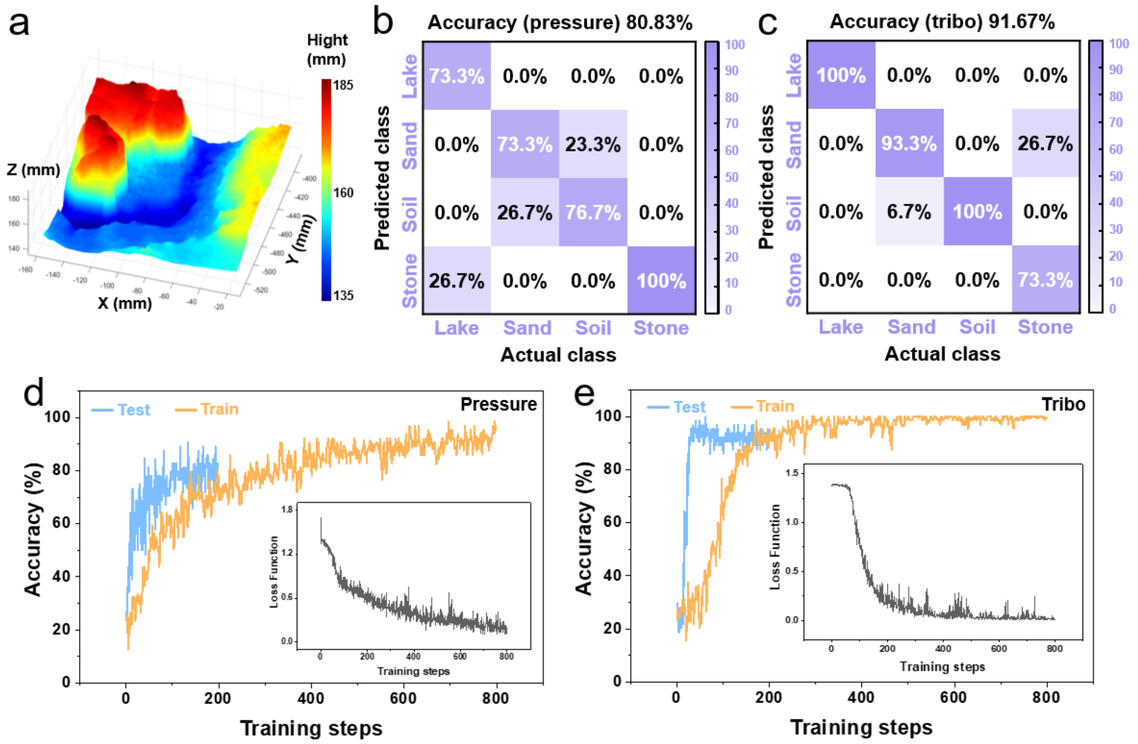

Fig. S26 Unimodal recognition results for Mars scenes. a) Three-dimensional topographic reconstruction map (pixel points: 5184) of the Mars micromodel, from which information on the undulation and height of the micromodel surface. **b**) Confusion matrix for pressure identification of Mars microscopic model. **c**) Confusion matrix for triboelectric identification of Mars microscopic model. **d**) Curve of pressure recognition accuracy with training steps. Inset: Curve of loss functional with training steps. **e**) Curve of triboelectric recognition accuracy with training steps. Inset: Curve of loss functional with training steps

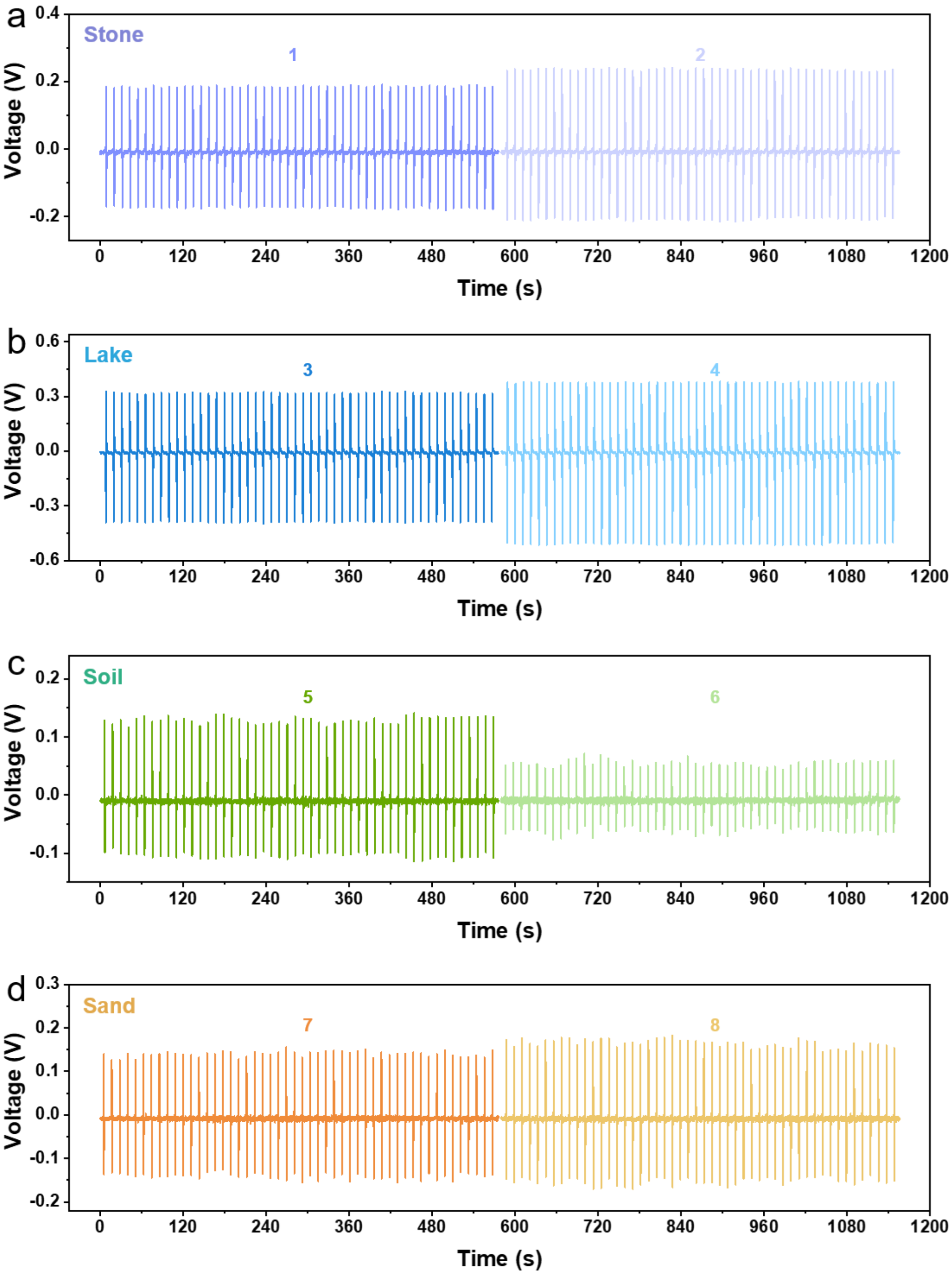

Fig. S27 Machine learning raw data of triboelectric. a) Triboelectric recognition signal of stone. **b**) Triboelectric recognition signal of lake. **c**) Triboelectric recognition signal of soil. **d**) Triboelectric recognition signal of sand

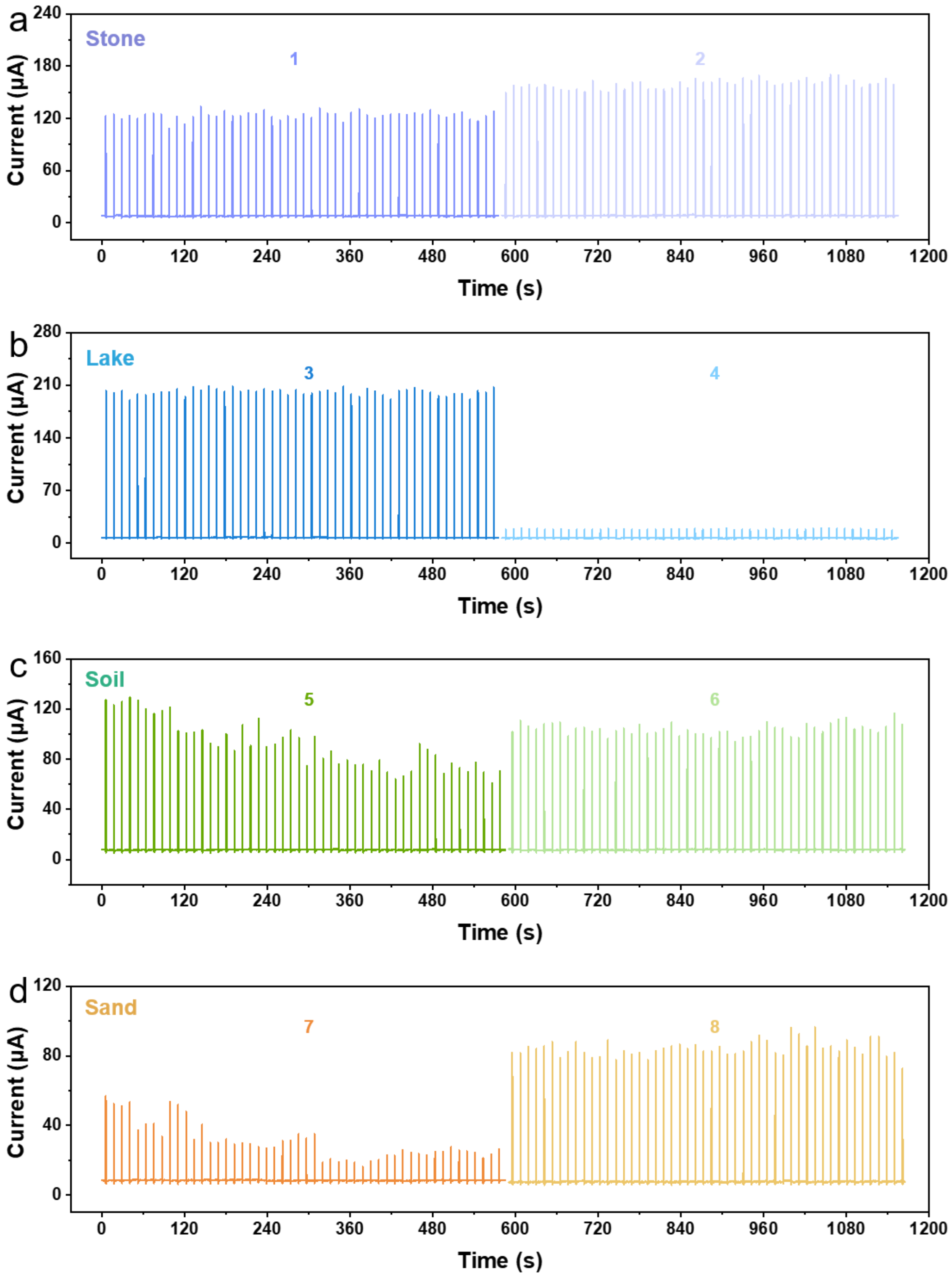

Fig. S28 Machine learning raw data of pressure. a) Pressure recognition signal of stone. **b**) Pressure recognition signal of lake. **c**) Pressure recognition signal of soil. **d**) Pressure recognition signal of sand

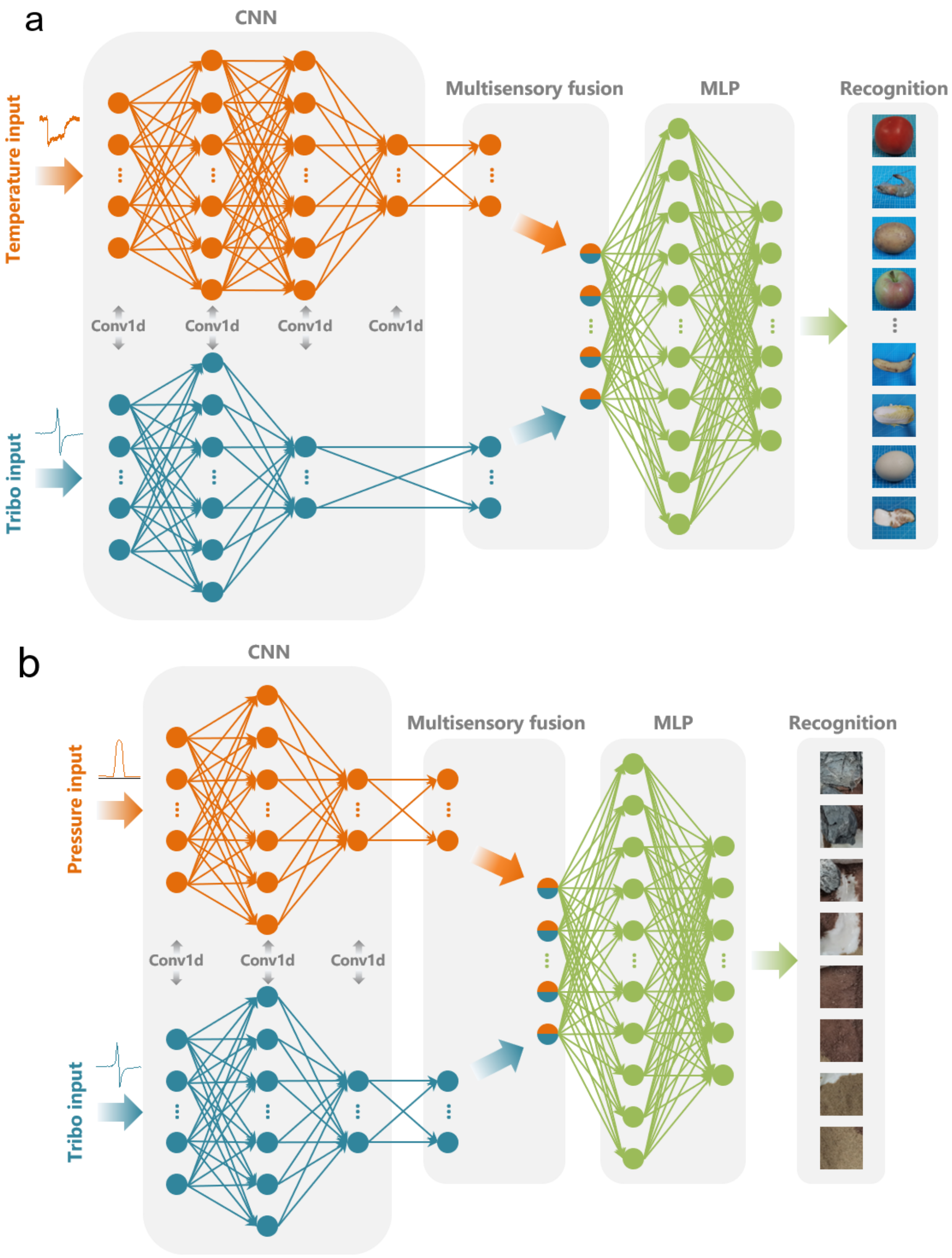

Fig. S29 a) Multimodal learning algorithm architecture for MTAS tactile system in kitchen scenario. **b**) Multimodal learning algorithm architecture for MTAS tactile system in Mars scenario

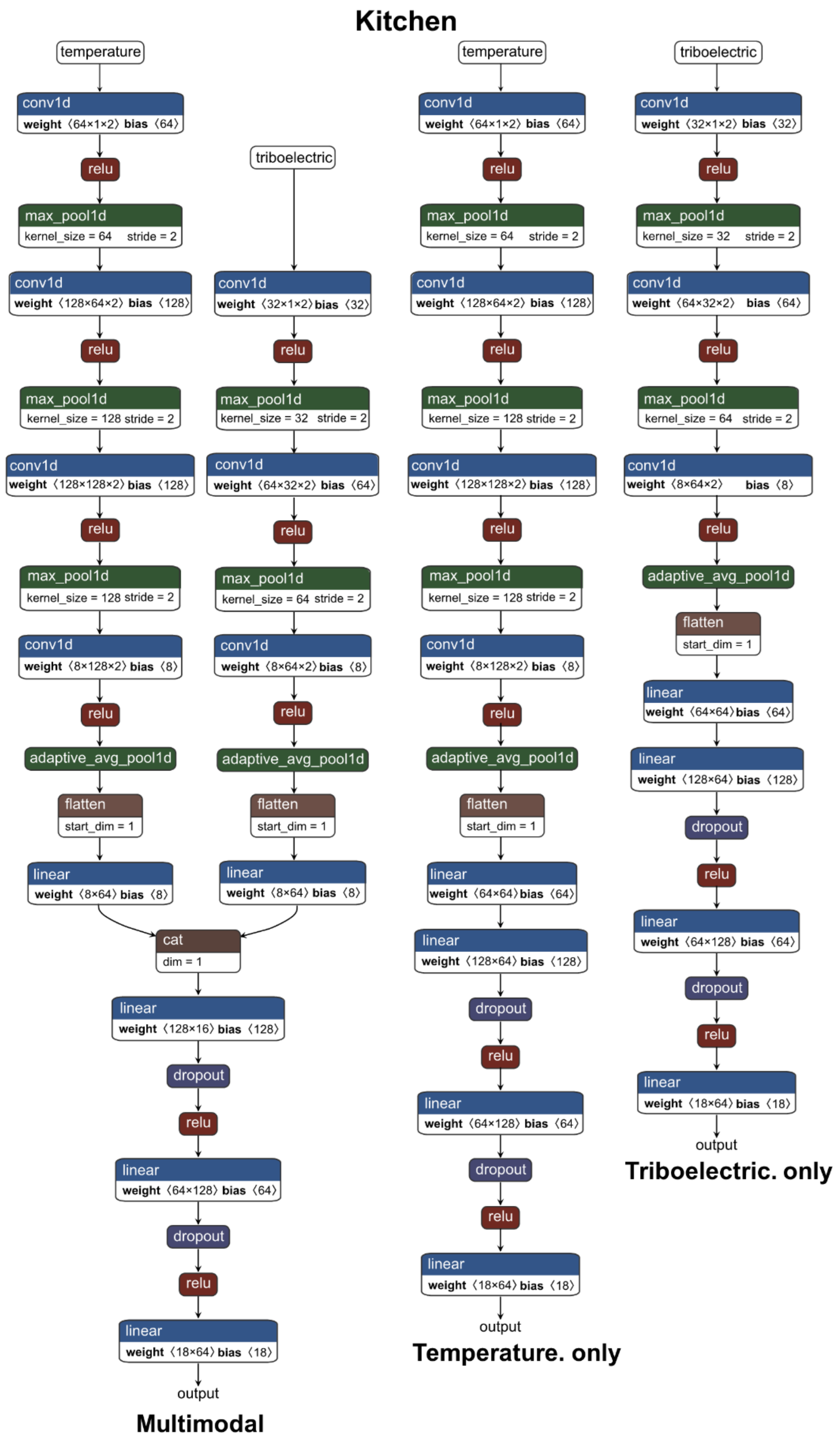

Fig. S30 Network Structure of the algorithm for the kitchen scene

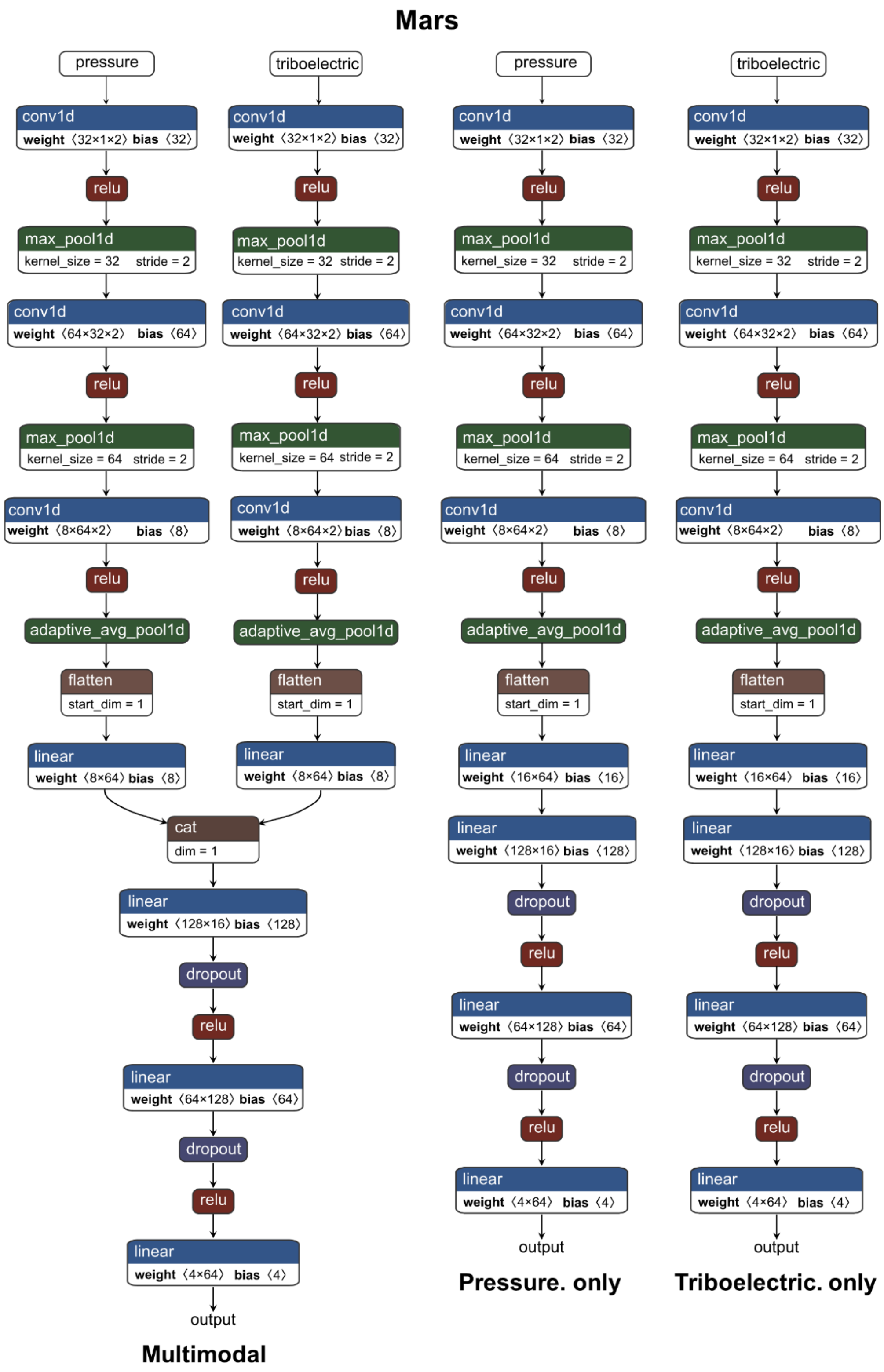

Fig. S31 Network Structure of the algorithm for the Mars scene

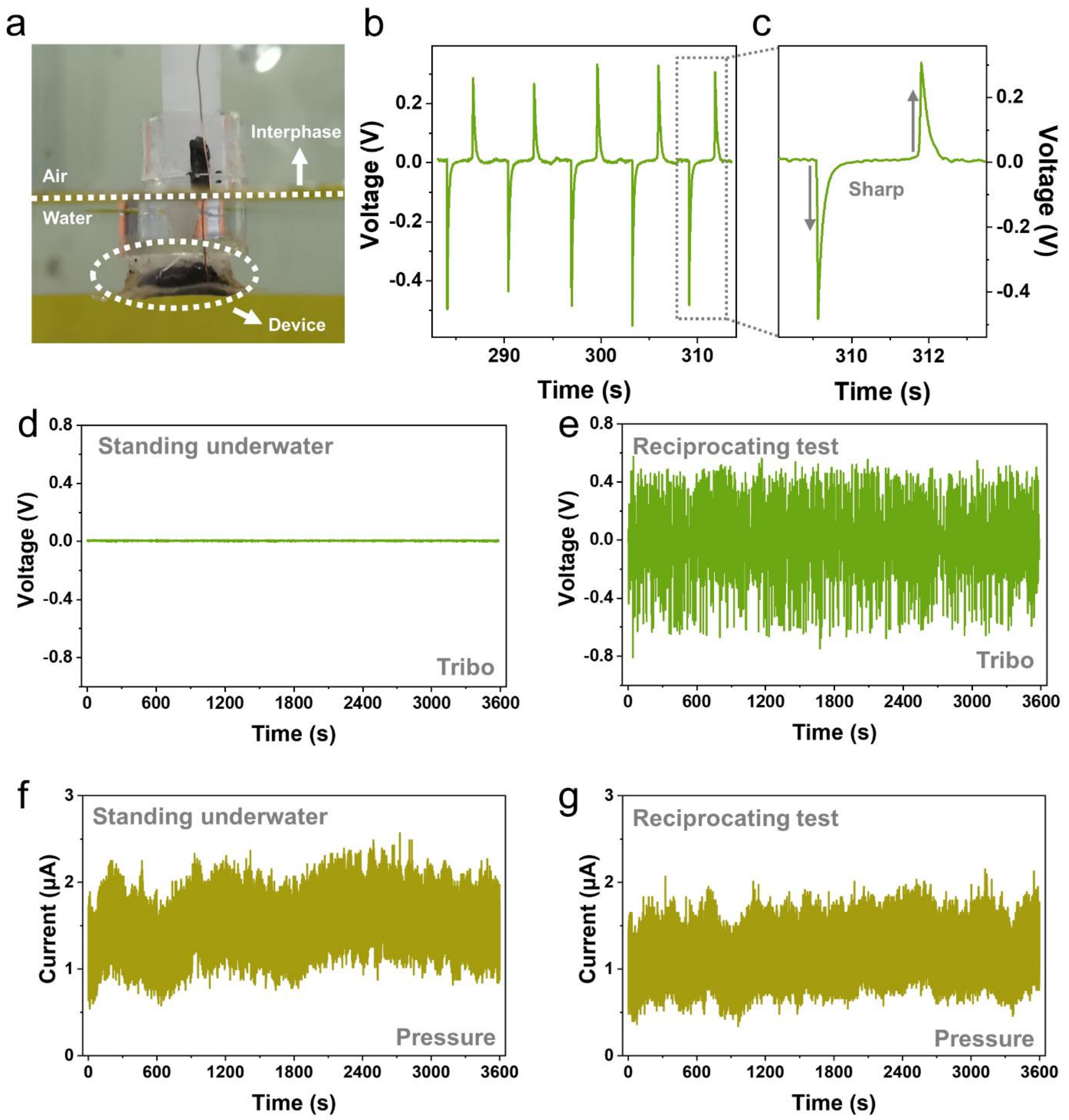

Fig. S32 Waterproof performance testing of MTAS. **a**) Schematic of the testing system. **b**) Output signal of MTAS reciprocating motion at the gas-liquid interface. **c**) Output signal for one cycle of MTAS reciprocating motion. **d**) Triboelectric signal of MTAS underwater standing for 1h. **e**) Triboelectric signal of MTAS reciprocating motion at the gas-liquid interface for 1h. **f**) Pressure signal of MTAS underwater standing for 1 h. **g**) Pressure signal of MTAS reciprocating motion at the gas-liquid interface for 1 h

Table S1 Sensing and object recognition ability comparison of multifunctional tactile systems in detail

Sensor type	Mechanism	Performance	Target	Algorithm	Multi-modal (input data)	Accuracy	Refs.
Pressure & temperature & biochemistry	Piezoresistive Thermal-sensitive Redox reaction	0-0.5 kPa 25-45 ℃ TNT, OP, biohazards (SARS-CoV-2 virus)	Gesture recognition (6 gestures) Contaminant recognition	KNN	× Gesture/Contaminant	97.29% -	[S12]

Pressure & olfactory	Piezoresistive Gas-sensitive(chemical reaction)	0.375 mV/kPa 0-400 kPa	Rescue recognition (11 objects)	BOT (CNN/FCN)	✓ Pressure+gas	96.9%	[S13]
Pressure & thermal conductivity & temperature	Piezoresistive Thermal-resistance	117 mV/kPa $0.0016℃^{-1}$	Object recognition (13 objects) Garbage classification (7 garbages)	MLP	✓ Pressure + thermal conductivity + object and environment temperature	96% 94%	[S14]
Pressure & vibration	Piezoresistive Piezoelectric	$0.01\ kPa^{-1}$ 0.1-100 kPa >1000 Hz	Texture recognition (20 fabrics)	ANN	✓ Pressure + vibration	99.1%	[S15]
Pressure & vibration	Piezoresistive Microphone	<20 N	Tactile type recognition (4 tactile)	CNN	× vibration	98.7%	[S16]
Band & temperature	Triboelectric Pyroelectric	0.3577V/℃	Sign language recognition (14 gestures) + Object recognition (5 objects)	SVM	× Triboelectric	99.821 % 94%	[S17]
Pressure & temperature & triboelectric	Piezoresistive Thermoelectric Charge transfer	$15.22\ kPa^{-1}$ 0-40 kPa 35.2 µV/K 25-60 °C	Materials recognition (10 objects)	lookup table	× Triboelectric	-	[S18]
**Pressure & temperature & triboelectric**	**Piezoresistive Thermoelectric Charge transfer**	**$92.22\ kPa^{-1}$ 0.04-100 kPa 11 ms 15.6 nA /°C 21.5-66.2 °C**	**Food recognition (18 food) + Mars recognition (4 landforms)**	**CNN + MLP**	✓ **Pressure + temperature + triboelectric**	**94.63% 100%**	**This work**

Table S2 List of hyperparameters and their values

Scence	Algorithm	hyperparameter	Value
Kitchen	Multimodal	batch size	64
		learning rate	5×10^{-4}
		weight decay	1×10^{-5}
		epochs number	200
	Triboelectricity. only	batch size	64
		learning rate	1×10^{-3}
		weight decay	1×10^{-5}
		epochs number	200
	Temperature. only	batch size	64
		learning rate	1×10^{-3}
		weight decay	1×10^{-5}
		epochs number	200

Mars	Multimodal	batch size	64
		learning rate	1×10^{-3}
		weight decay	1×10^{-5}
		epochs number	200
	Triboelectricity. only	batch size	64
		learning rate	1×10^{-3}
		weight decay	1×10^{-5}
		epochs number	200
	Pressure. only	batch size	64
		learning rate	1×10^{-3}
		weight decay	1×10^{-5}
		epochs number	200

Notes:
batch size: Number of training cases over which each Adam update is computed
learning rate: The learning rate used by Adam
weight decay: The weight decay used by Adam
epochs number: Number of full passes of the entire training dataset through the network training

Supplementary References

[S1] B. E. Stein, T. R. Stanford, Multisensory integration: current issues from the perspective of the single neuron. Nat. Rev. Neurosci. **9**, 255-266 (2008). https://doi.org/10.1038/nrn2331

[S2] N. Fazeli, M. Oller, J. Wu, Z. Wu, J. B. Tenenbaum, A. Rodriguez, See, feel, act: Hierarchical learning for complex manipulation skills with multisensory fusion. Sci. Robot. **4**, eaav3123 (2019). https://doi.org/10.1126/scirobotics.aav3123

[S3] J. Pesnot Lerousseau, C. V. Parise, M. O. Ernst, V. van Wassenhove, Multisensory correlation computations in the human brain identified by a time-resolved encoding model. Nat. Commun. **13**, 2489 (2022). https://doi.org/10.1038/s41467-022-29687-6

[S4] H. Tan, Y. Zhou, Q. Tao, J. Rosen, S. van Dijken, Bioinspired multisensory neural network with crossmodal integration and recognition. Nat. Commun. **12**, 1120 (2021). https://doi.org/10.1038/s41467-021-21404-z

[S5] A. Billard, D. Kragic, Trends and challenges in robot manipulation. Science **364**, eaat8414 (2019). https://doi.org/10.1126/science.aat8414

[S6] H. Sun, K. J. Kuchenbecker, G. Martius, A soft thumb-sized vision-based sensor with accurate all-round force perception. Nat. Mach. Intell. **4**, 135-145 (2022). https://doi.org/10.1038/s42256-021-00439-3

[S7] M. Wang, Z. Yan, T. Wang, P. Cai, S. Gao et al., Gesture recognition using a bioinspired learning architecture that integrates visual data with somatosensory data from stretchable sensors. Nat. Electron. **3**, 563-570 (2020). https://doi.org/10.1038/s41928-020-0422-z

[S8] P. Dudek, T. Richardson, L. Bose, S. Carey, J. Chen, C. Greatwood, et al., Sensor-level computer vision with pixel processor arrays for agile robots. Sci. Robot. **7**, eabl7755 (2022). https://doi.org/10.1126/scirobotics.abl7755

[S9] B. Peters, N. Kriegeskorte, Capturing the objects of vision with neural networks. Nat. Hum. Behav. **5**, 1127-1144 (2021). https://doi.org/10.1038/s41562-021-01194-6

[S10] K. Nasr, P. Viswanathan, A. Nieder, Number detectors spontaneously emerge in a deep neural network designed for visual object recognition. Sci. Adv. **5**, eaav7903 (2019). https://doi.org/10.1126/sciadv.aav7903

[S11] M. Liu, Y. Zhang, J. Wang, N. Qin, H. Yang et al., A star-nose-like tactile-olfactory bionic sensing array for robust object recognition in non-visual environments. Nat. Commun. **13**, 79 (2022). https://doi.org/10.1038/s41467-021-27672-z

[S12] Y. Yu, J. Li, A. Solomon Samuel, J. Min, J. Tu et al., All-printed soft human-machine interface for robotic physicochemical sensing. Sci. Robot. **7**, eabn0495 (2022). https://doi.org/10.1126/scirobotics.abn0495

[S13] Y. Wang, H. Wu, L. Xu, H. Zhang, Y. Yang et al., Hierarchically patterned self-powered sensors for multifunctional tactile sensing. Sci. Adv. **6**, (2020). https://doi.org/10.1126/sciadv.abb9083

[S14] K. Park, H. Yuk, M. Yang, J. Cho, H. Lee et al., A biomimetic elastomeric robot skin using electrical impedance and acoustic tomography for tactile sensing. Sci. Robot. **7**, eabm7187 (2022). https://doi.org/10.1126/scirobotics.abm7187

[S15] Y. Luo, Y. Li, P. Sharma, W. Shou, K. Wu et al., Learning human–environment interactions using conformal tactile textiles. Nat. Electron. **4**, 193-201 (2021). https://doi.org/10.1038/s41928-021-00558-0

[S16] Z. Sun, M. Zhu, X. Shan, C. Lee, Augmented tactile-perception and haptic-feedback rings as human-machine interfaces aiming for immersive interactions. Nat. Commun. **13**, 5224 (2022). https://doi.org/10.1038/s41467-022-32745-8

[S17] S. Chun, J.-S. Kim, Y. Yoo, Y. Choi, S. J. Jung et al., An artificial neural tactile sensing system. Nat. Electron. **4**, 429-438 (2021). https://doi.org/10.1038/s41928-021-00585-x

[S18] G. Li, S. Liu, L. Wang, R. Zhu, Skin-inspired quadruple tactile sensors integrated on a robot hand enable object recognition. Sci. Robot. **5**, eabc8134 (2020). https://doi.org/10.1126/scirobotics.abc8134

[S19] Y. Roh, M. Kim, S. M. Won, D. Lim, I. Hong et al., Vital signal sensing and manipulation of a microscale organ with a multifunctional soft gripper. Sci. Robot. **6**, eabi6774 (2021). https://doi.org/10.1126/scirobotics.abi6774

[S20] Y. Gao, C. Yan, H. Huang, T. Yang, G. Tian et al., Microchannel-Confined MXene Based Flexible Piezoresistive Multifunctional Micro-Force Sensor. Adv. Funct. Mater. **30**, 1909603 (2020). https://doi.org/10.1002/adfm.201909603

[S21] B. Wang, X. Lai, H. Li, C. Jiang, J. Gao et al., Multifunctional MXene/Chitosan-Coated Cotton Fabric for Intelligent Fire Protection. ACS Appl. Mater. Interfaces **13**, 23020-23029 (2021). https://doi.org/10.1021/acsami.1c05222

[S22] L. Groo, D. J. Inman, H. A. Sodano, In Situ Damage Detection for Fiber-Reinforced Composites Using Integrated Zinc Oxide Nanowires. Adv. Funct. Mater. **28**, 1802846 (2018). https://doi.org/10.1002/adfm.201802846

[S23] J. Wen, J. Tang, H. Ning, N. Hu, Y. Zhu et al., Multifunctional Ionic Skin with Sensing, UV-Filtering, Water-Retaining, and Anti-Freezing Capabilities. Adv. Funct. Mater. **31**, 2011176 (2021). https://doi.org/10.1002/adfm.202011176

[S24] H. Zhuo, Y. Hu, X. Tong, Z. Chen, L. Zhong et al., A Supercompressible, Elastic, and Bendable Carbon Aerogel with Ultrasensitive Detection Limits for Compression Strain, Pressure, and Bending Angle. Adv. Mater. **30**, 1706705 (2018). https://doi.org/10.1002/adma.201706705

[S25] Z. Sun, M. Zhu, Z. Zhang, Z. Chen, Q. Shi et al., Artificial Intelligence of Things (AIoT) Enabled Virtual Shop Applications Using Self-Powered Sensor Enhanced Soft Robotic Manipulator. Adv. Sci. **8**, 2100230 (2021). https://doi.org/10.1002/advs.202100230

[S26] C. M. Boutry, M. Negre, M. Jorda, O. Vardoulis, A. Chortos et al., A hierarchically patterned, bioinspired e-skin able to detect the direction of applied pressure for robotics. Sci. Robot. **3**, eaau6914 (2018). https://doi.org/10.1126/scirobotics.aau6914

[S27] O. Feinerman, I. Pinkoviezky, A. Gelblum, E. Fonio, N. S. Gov, The physics of cooperative transport in groups of ants. Nat. Phys. **14**, 683-693 (2018). https://doi.org/10.1038/s41567-018-0107-y

[S28] T. Li, A. D. Pickel, Y. Yao, Y. Chen, Y. Zeng et al., Thermoelectric properties and performance of flexible reduced graphene oxide films up to 3,000 K. Nat. Energy **3**, 148-156 (2018). https://doi.org/10.1038/s41560-018-0086-3

[S29] Z. Wei, D. Wang, S. Kim, S.-Y. Kim, Y. Hu et al., Nanoscale Tunable Reduction of Graphene Oxide for Graphene Electronics. Science **328**, 1373-1376 (2010). https://doi.org/10.1126/science.1188119

[S30] I. You, D. G. Mackanic, N. Matsuhisa, J. Kang, J. Kwon et al., Artificial multimodal receptors based on ion relaxation dynamics. Science **370**, 961-965 (2020). https://doi.org/10.1126/science.aba5132

[S31] J. Park, M. Kim, Y. Lee, H. S. Lee, H. Ko, Fingertip skin–inspired microstructured ferroelectric skins discriminate static/dynamic pressure and temperature stimuli. Sci. Adv. **1**, e1500661 (2015). https://doi.org/10.1126/sciadv.1500661

[S32] C. Chen, Y. Kuang, S. Zhu, I. Burgert, T. Keplinger et al., Structure–property–function relationships of natural and engineered wood. Nat. Rev. Mater. **5**, 642-666 (2020). https://doi.org/10.1038/s41578-020-0195-z

[S33] H. Zou, Y. Zhang, L. Guo, P. Wang, X. He et al., Quantifying the triboelectric series. Nat. Commun. **10**, 1427 (2019). https://doi.org/10.1038/s41467-019-09461-x

[S34] Z. L. Wang, Triboelectric Nanogenerators as New Energy Technology for Self-Powered Systems and as Active Mechanical and Chemical Sensors. Acs Nano **7**, 9533-9557 (2013). https://doi.org/10.1021/nn404614z

[S35] Z. L. Wang, On Maxwell's displacement current for energy and sensors: the origin of nanogenerators. Mater. Today **20**, 74-82 (2017). https://doi.org/10.1016/j.mattod.2016.12.001

[S36] X. Qu, Z. Liu, P. Tan, C. Wang, Y. Liu et al., Artificial tactile perception smart finger for material identification based on triboelectric sensing. Sci. Adv. **8**, eabq2521 (2022). https://doi.org/10.1126/sciadv.abq2521

[S37] C. Leovy, Weather and climate on Mars. Nature **412**, 245-249 (2001). https://doi.org/10.1038/35084192

[S38] L. K. Fenton, P. E. Geissler, R. M. Haberle, Global warming and climate forcing by recent albedo changes on Mars. Nature **446**, 646-649 (2007). https://doi.org/10.1038/nature05718

[S39] A. S. Yen, R. Gellert, C. Schröder, R. V. Morris, J. F. Bell et al., An integrated view of the chemistry and mineralogy of martian soils. Nature **436**, 49-54 (2005). https://doi.org/10.1038/nature03637

[S40] R. Rieder, T. Economou, H. Wänke, A. Turkevich, J. Crisp et al., The Chemical Composition of Martian Soil and Rocks Returned by the Mobile Alpha Proton X-ray Spectrometer: Preliminary Results from the X-ray Mode. Science **278**, 1771-1774 (1997). https://doi.org/10.1126/science.278.5344.1771

[S41] D. Clery, Lake spied deep below polar ice cap on Mars. Science **361**, 320-320 (2018). https://doi.org/10.1126/science.361.6400.320

[S42] A. Diez, Liquid water on Mars. Science **361**, 448-449 (2018). https://doi.org/10.1126/science.aau1829

[S43] R. Orosei, S. E. Lauro, E. Pettinelli, A. Cicchetti, M. Coradini et al., Radar evidence of subglacial liquid water on Mars. Science **361**, 490-493 (2018). https://doi.org/10.1126/science.aar7268

[S44] S. E. Lauro, E. Pettinelli, G. Caprarelli, L. Guallini, A. P. Rossi et al., Multiple subglacial water bodies below the south pole of Mars unveiled by new MARSIS data. Nat. Astron. **5**, 63-70 (2021). https://doi.org/10.1038/s41550-020-1200-6

[S45] Y. Liu, X. Wu, Y.-Y. S. Zhao, L. Pan, C. Wang et al., Zhurong reveals recent aqueous activities in Utopia Planitia, Mars. Sci. Adv. **8**, eabn8555 (2022). https://doi.org/10.1126/sciadv.abn8555

[S46] H.-L. Gao, Y.-B. Zhu, L.-B. Mao, F.-C. Wang, X.-S. Luo et al., Super-elastic and fatigue resistant carbon material with lamellar multi-arch microstructure. Nat. Commun. **7**, 12920 (2016). https://doi.org/10.1038/ncomms12920

[S47] X. Shi, X. Fan, Y. Zhu, Y. Liu, P. Wu et al., Pushing detectability and sensitivity for subtle force to new limits with shrinkable nanochannel

structured aerogel. Nat. Commun. **13**, 1119 (2022). https://doi.org/10.1038/s41467-022-28760-4

[S48] H. Zhuo, Y. Hu, Z. Chen, X. Peng, L. Liu et al., A carbon aerogel with super mechanical and sensing performances for wearable piezoresistive sensors. J. Mater. Chem. A **7**, 8092-8100 (2019). https://doi.org/10.1039/c9ta00596j

[S49] X. Peng, K. Wu, Y. Hu, H. Zhuo, Z. Chen et al., A mechanically strong and sensitive CNT/rGO–CNF carbon aerogel for piezoresistive sensors. J. Mater. Chem. A **6**, 23550-23559 (2018). https://doi.org/10.1039/c8ta09322a

[S50] C. Xu, Y. Zi, A. C. Wang, H. Zou, Y. Dai et al., On the Electron-Transfer Mechanism in the Contact-Electrification Effect. Adv. Mater. **30**, 1706790 (2018). https://doi.org/10.1002/adma.201706790

[S51] Z. L. Wang, A. C. Wang, On the origin of contact-electrification. Mater. Today **30**, 34-51 (2019). https://doi.org/10.1016/j.mattod.2019.05.016
